# Tissue- and Liquid-Based Biomarkers in Prostate Cancer Precision Medicine

**DOI:** 10.3390/jpm11070664

**Published:** 2021-07-15

**Authors:** James Meehan, Mark Gray, Carlos Martínez-Pérez, Charlene Kay, Duncan McLaren, Arran K. Turnbull

**Affiliations:** 1 Translational Oncology Research Group, Institute of Genetics and Cancer, Western General Hospital, University of Edinburgh, Edinburgh EH4 2XU, UK; carlos.martinez-perez@ed.ac.uk (C.M.-P.); charlene.kay@ed.ac.uk (C.K.); a.turnbull@ed.ac.uk (A.K.T.); 2 The Royal (Dick) School of Veterinary Studies and Roslin Institute, University of Edinburgh, Midlothian EH25 9RG, UK; mark.gray@ed.ac.uk; 3 Breast Cancer Now Edinburgh Research Team, Institute of Genetics and Cancer, Western General Hospital, University of Edinburgh, Edinburgh EH4 2XU, UK; 4 Edinburgh Cancer Centre, Western General Hospital, NHS Lothian, Edinburgh EH4 2XU, UK; duncan.mclaren@nhslothian.scot.nhs.uk

**Keywords:** prostate cancer, precision medicine, tissue-based biomarkers, liquid-based biomarkers

## Abstract

Worldwide, prostate cancer (PC) is the second-most-frequently diagnosed male cancer and the fifth-most-common cause of all cancer-related deaths. Suspicion of PC in a patient is largely based upon clinical signs and the use of prostate-specific antigen (PSA) levels. Although PSA levels have been criticised for a lack of specificity, leading to PC over-diagnosis, it is still the most commonly used biomarker in PC management. Unfortunately, PC is extremely heterogeneous, and it can be difficult to stratify patients whose tumours are unlikely to progress from those that are aggressive and require treatment intensification. Although PC-specific biomarker research has previously focused on disease diagnosis, there is an unmet clinical need for novel prognostic, predictive and treatment response biomarkers that can be used to provide a precision medicine approach to PC management. In particular, the identification of biomarkers at the time of screening/diagnosis that can provide an indication of disease aggressiveness is perhaps the greatest current unmet clinical need in PC management. Largely through advances in genomic and proteomic techniques, exciting pre-clinical and clinical research is continuing to identify potential tissue, blood and urine-based PC-specific biomarkers that may in the future supplement or replace current standard practices. In this review, we describe how PC-specific biomarker research is progressing, including the evolution of PSA-based tests and those novel assays that have gained clinical approval. We also describe alternative diagnostic biomarkers to PSA, in addition to biomarkers that can predict PC aggressiveness and biomarkers that can predict response to certain therapies. We believe that novel biomarker research has the potential to make significant improvements to the clinical management of this disease in the near future.

## 1. Introduction

Prostate cancer (PC) was first reported in 1853 after a histological examination conducted by Dr. J. Adams, a surgeon in The London Hospital [1]. Adams noted in his description that it was ‘a very rare disease’, a comment that now contrasts greatly to how significant PC has become in the field of oncology. Worldwide, PC is the second-most-frequently diagnosed male cancer and the fifth-most-common cause of cancer-related mortalities. Current estimates indicate that ~1.4 million new cases are diagnosed and 400,000 PC-related deaths occur every year [2]. In the United States of America PC alone accounts for 26% of cancer diagnoses in men [3], while recently in the United Kingdom PC has overtaken breast cancer to become the most commonly diagnosed cancer [4]. PC is more frequently identified in elderly men, with estimates indicating that ~60% of cases are diagnosed in those older than 65 years of age [5]. Due to the aging nature of the global population, it is thought that the social and economic impact of PC will increase significantly over the coming years.

### Prostate Cancer: Risk Classification, Treatment and Challenges

PC is a highly heterogeneous disease with widely varying clinical outcomes. Some PC patients present with slow growing, localised cancers that do not pose an immediate risk to overall health. These patients may never go onto develop clinical symptoms, and in the absence of screening programmes they would never have known that they had PC [6]. Other tumours, however, can grow rapidly, resist treatment, metastasise early and can be fatal. Knowledge of PC aggressiveness is very important in determining the most appropriate treatment for each patient. Current methods for stratifying patient risk involve (i) staging, i.e., determining the extent of the tumour in the body. (ii) Gleason grading, an indication of cancer aggressiveness based on the architecture or pattern of the glands within the prostate. Scores range from 1 to 5, with the most common and second most predominant scores combined to give the final Gleason score (low grade = 6, intermediate grade = 7, high grade ≥ 8). (iii) The assessment of prostate specific antigen (PSA) levels. Together, this information is used to determine whether a patient is within a low-, intermediate- or high-risk group [7]. Typically, tumours will be histologically graded using needle core biopsy tissue prior to the patient starting treatment. As Gleason grade continues to be regarded as the greatest predictor of prognosis [8], there is a universal dependence on biopsy samples for risk assessment and treatment selection. However, there are many significant limitations associated with the use of tissue biopsies. PC is different to many other tumour types in that at the time of diagnosis, 60–90% of patients have multiple, separate and potentially diverse cancer foci scattered throughout the prostate. These foci can develop independently and can differ in their aggressiveness [9]. Thus, from a treatment perspective, tumour heterogeneity represents a significant challenge for biopsy-based assays to determine PC aggressiveness, as it can lead to differences in the grade observed between the diagnostic biopsy specimen and the final grade based upon samples acquired following surgery [10]. From the patient’s perspective, the acquisition of tissue biopsies is an invasive procedure and can lead to side effects that include rectal bleeding, haematuria, infection and pain [11].

There are a variety of treatment options available to newly diagnosed PC patients [12]. Active surveillance (AS) is one option for low- or favourable-intermediate risk patients; this involves regular testing to assess whether or not their cancer is growing or spreading. If there are signs of disease progression, or if a patient is deemed higher risk, then definitive treatments including radical prostatectomy (RP), radiotherapy (RT) and androgen deprivation therapy (ADT) can be provided. About 87% of patients diagnosed with localised PC are given some form of radical therapy [13]. Unfortunately, treatments such as RT and RP can lead to substantial complications (including urinary, bowel and sexual dysfunction), each of which can significantly affect patient quality of life [14,15].

The focus of PC-specific biomarker research has previously been on disease diagnosis. There is, however, an increasing clinical need for the identification of novel prognostic, predictive and treatment response biomarkers that can be used to provide a precision medicine approach to PC management. Due to the significant complications associated with definitive treatment, the identification of biomarkers at the time of screening/diagnosis that provide an indication of the risk of aggressiveness is perhaps currently the greatest unmet clinical need in PC management. Biomarkers that help fulfil this role would help clinicians determine the most appropriate treatment strategy for newly diagnosed patients (i.e., who should be considered for AS and who should undergo radical treatment).

**Figure 1:**
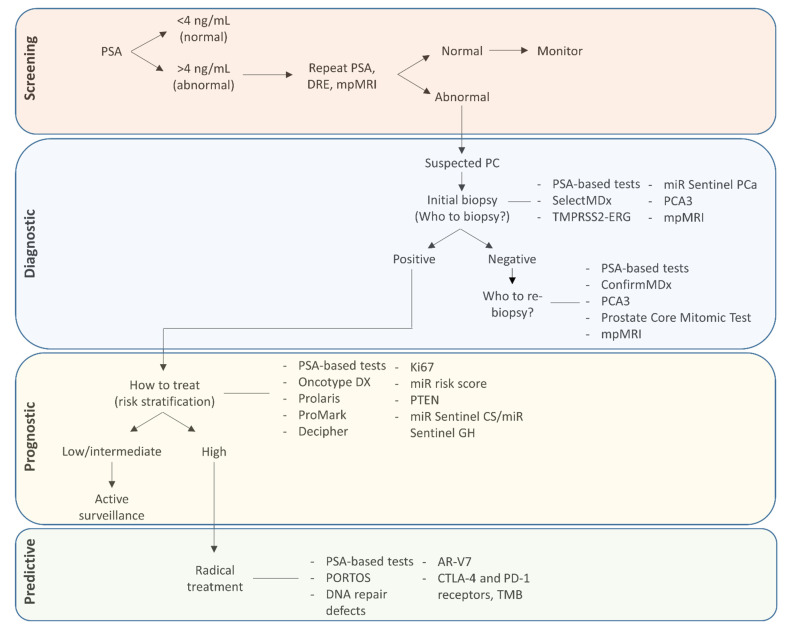
Biomarker assays and their use in PC management. AR-V7, androgen receptor splice variant 7; CTLA-4, cytotoxic T lymphocyte-associated protein-4; DRE, Digital rectal examination; mpMRI, multi-parametric magnetic resonance imaging; PC, Prostate cancer; PD-1, programmed death-1; PORTOS, Post-Operative Radiation Therapy Outcomes Score; PSA, Prostate specific antigen; PTEN, phosphatase and tensin homolog; TMB, Tumour mutational burden.

Largely through advances in genomic and proteomic techniques, exciting pre-clinical and clinical research is continuing to identify potential tissue, blood and urine-based PC-specific biomarkers that may in the future supplement or replace current standard practices. This review will provide an overview of selected biomarkers that have the potential to increase the likelihood of PC detection, reduce over-diagnosis, predict the risk of progression and recurrence, and also give an indication of treatment response. We will begin by discussing PSA, which is unique as a biomarker as it has can be used for both PC detection, prognosis and to assess the effects of treatment. We will then go onto to discuss other biomarkers that have a role in the pre-diagnostic and post-diagnostic settings. Figure 1 and Table 1 outline where and how each of the biomarkers and their associated tests, discussed in this review, can contribute to PC patient management.

## 2. Prostate Specific Antigen

Prostate specific antigen (PSA) is a blood-based biomarker that can be used in the screening of patients for PC detection, in the surveillance of patients following diagnosis, to assess the risk of PC recurrence, and for monitoring treatment responses. PSA, a kallekrein-like serine protease glycoprotein, is encoded by the prostate-specific gene kallikrein 3 (*KLK3*) [16]. PSA is secreted by prostatic epithelial cells, with low levels of this glycoprotein typically present in blood samples from healthy individuals. Its primary function is to liquefy semen through proteolysis [16]. Although the specific mechanisms are open to debate, raised PSA levels within the blood of men with PC are not due to amplified expression of the protein, but instead result from increased release of PSA into the blood due to the disruption of prostate architecture observed in prostate tumours [17]. While there is no recognised defined cut-off for diagnosing PC, many clinicians consider PSA levels ≤ 4.0 ng/mL as normal, with higher levels indicating a need for further investigation. PC patients with unexpectedly high PSA levels have been encountered, with concentrations as high as 23,126 ng/mL previously reported [18]. 

### 2.1. PSA and Screening

In the first large scale investigation of the clinical use of PSA, levels of this protein were found to be associated with the clinical stage of PC, with increased levels correlated with more advanced disease stages [19]. Later studies investigated the use of PSA in terms of its ability to screen the population for disease, with a view to detecting early-stage PC. These reports highlighted that, when used in conjunction with clinical findings, PSA levels of ≥4.0 ng/mL resulted in improved PC detection [20,21,22]. The assessment of PSA levels was approved by the US Food and Drug Administration (FDA) as a diagnostic tool for PC detection in 1994 [23]. Its use as a screening test among asymptomatic men gained popularity, which in the US alone led to a dramatic increase in PC incidence [24]. The proportion of patients diagnosed at first presentation with metastatic disease also reduced following its use in screening programmes [24]. However, a significant criticism of the widespread use of PSA testing in the population was that it led to a PC diagnosis in men that would never have otherwise been diagnosed with clinically significant PC; the term “over-diagnosis” is often used to describe this situation [24]. Over-diagnosis, in relation to PSA screening programmes, has been reported to range from 20–66% [25,26]. Decreasing the number of PC patients diagnosed with later stage disease, while also increasing the number of patients receiving treatment, led to concerns that PC had become over-treated [27]. As well as having cost implications, over-treating PC can have significant effects on the mental and physical health of patients. As previously mentioned, even diagnostic procedures such as a prostatic biopsy carry risks of complications [11], while the side effects from RP and RT, which can occur in 50% of patients, can be severe. Recognised side effects from these treatments include urinary incontinence, sexual dysfunction and diminished colonic/rectal function [28,29].

Unfortunately, there is still debate on the extent to which PSA screening decreased PC mortality rates observed in the 1990s. The Cluster Randomised Trial of PSA Testing for Prostate Cancer (CAP) [30], the European Randomised Study of Screening for Prostate Cancer (ERSPC) [31,32] and the Prostate, Lung, Colorectal, and Ovarian Cancer Screening Trial (PLCO) [33] were three large randomised prospective trials that assessed the value of PSA screening in asymptomatic men for PC diagnosis. While the ERSPC trial found that screening for PC lowered PC-specific mortality and reduced the risk of developing metastatic disease, the two other trials did not replicate these results. Even though each of these trials assessed asymptomatic men between 50–60 years old, the trials differed greatly in their design, with limitations associated with each of them (for example, a screening/no-screening comparison was not strictly performed in the PLCO trial, as up to 90% of those in the “control group” had at least one PSA test, either before the screening began or over the course of the screening period) [34]. A recent systematic review for the US Preventive Services Task Force (USPSTF) suggested that PSA screening does have the ability to lower PC mortality risk, but it is linked with false-positive results, complications from resulting biopsy procedures and over-diagnosis [26]. 

In 2018, the USPSTF stated that results from screening trials had failed to show reductions in all-cause mortality and that there was inadequate evidence to suggest a benefit from PSA screening to decrease PC mortality in men over the age of 70. They also concluded that the net benefit of PSA-based screening for PC in men between 55–69 years is small [28]. As a result of the uncertainty over the benefits of PSA in screening, most of the guidelines that have been published are against mass screening, but advocate screening in men over 50 years of age with greater than 10 years life expectancy, only after the potential benefits and harms of screening have been outlined to the patient [28,35,36,37]. In contrast, the European Association of Urology and the Memorial Sloan Kettering Cancer Center both recommend that PSA screening should begin in well-informed men at 45 years of age, with the interval of testing thereafter dependent on the levels observed in this first test [38,39].

### 2.2. PSA and Prognosis

As well as having use in patient screening, PSA levels can also be utilised to estimate prognosis in newly diagnosed PC patients. In general, the more elevated the PSA levels are, the poorer the outcome [40,41,42,43,44]. Studies have shown that PSA levels > 20 ng/mL at diagnosis lead to a significant decrease in 5-year survival rates, with PSA concentrations above 98 ng/mL leading to a greater than 50% decrease in survival. The authors concluded that these highly elevated PSA concentrations suggest the presence of more aggressive or occult metastatic disease, thus indicating that these patients might benefit from more aggressive treatments [42]. While this relationship between high PSA levels and poor prognosis is especially relevant in PC patients with low or intermediate grade PC, in patients with high grade disease (Gleason score 8–10), lower PSA levels can actually predict a poorer outcome [45,46]; 10% of PC patients with higher grade disease had PSA readings of ≤2.5 ng/mL [46]. Additionally, reports have indicated that patients who present with PSA levels lower than 4 ng/mL have a greater incidence of distant metastasis than those with PSA concentrations between 4–10 ng/mL, 10–20 ng/mL or  >20 ng/mL; Zheng et al. inferred that clinicians should pay particular attention to those patients with lower PSA levels, as their disease may be biologically aggressive [43]. Even though there is a correlation between PSA levels at diagnosis and outcome, PSA has only limited prognostic accuracy when utilised alone. To improve prognostic accuracy within the clinic, tumour histological and clinical factors are assessed alongside PSA levels when predicting outcome [34].

### 2.3. Use of PSA Following Initial Diagnosis

There are a variety of management options available to patients with newly diagnosed PC. Regardless of the therapy chosen, PSA levels are commonly analysed following the instigation of initial definitive treatment(s). The optimal frequency of PSA testing has yet to be ascertained. After definitive therapy, PSA testing is advised every 6–12 months for the first 5 years, which can then subsequently be reduced to once a year. PSA testing may be carried out more regularly in those patients that are at a higher risk of recurrence (Gleason score 8–10 or PSA > 20 ng/mL) [34]. PSA concentrations observed after therapy differ depending on the treatment given. Within 2 months of RP in patients with localised PC, PSA concentrations generally decrease to undetectable levels (<0.1 ng/mL) [34]. Two successive increasing PSA measurements of >0.2 ng/mL is defined as biochemical recurrence (BCR) after RP [47]. PSA concentrations reduce more slowly after RT or brachytherapy, with concentrations of <0.5 ng/mL generally observed 6 months after treatment. Transient increases in PSA levels may also occur post-RT within 3 years after treatment [48,49]. Increases in PSA levels of 2 ng/mL or more above the PSA nadir (also known as the Phoenix definition of BCR [50]) is regarded as BCR after RT [51].

The clinical management of patients that exhibit BCR after primary treatment is a controversial issue [52]. Even though BCR signifies a higher risk of clinical recurrence, many men remain symptom-free after its manifestation. In one study, only 34% of patients that exhibited BCR later showed signs of clinical recurrence. In those that did suffer recurrence, 8 years was the median duration of time between BCR and metastasis, with an additional median time to death of 5 years [53]. Clinicians therefore face the challenge of preventing or delaying progression in those patients that are deemed to be at risk, while also avoiding the over-treatment of men whose disease might never continue past PSA-only recurrence. There have been attempts to distinguish factors linking BCR to the risk of clinical recurrence; higher Gleason scores and shorter intervals to BCR have been associated with recurrence risk after both RP and RT [54].

Because many patients that exhibit BCR never go on to develop signs of clinical recurrence, there is still debate on whether ADT should be given early, or if clinicians should delay administration until clinical evidence of disease recurrence is present [55,56]. While an initial study comparing immediate ADT (patients treated within 3 months of PSA relapse) to deferred ADT (patients treated when they presented with clinical symptoms) demonstrated that there was no difference in 5 year overall survival rates between the two groups [57], more recent work indicates that prompt treatment with ADT may lead to better outcomes [58]. PSA kinetics and time to PSA nadir are important indicators of response to primary ADT treatment. However, the prognostic significance of PSA kinetics after primary ADT continues to be controversial. Intuitively, many urologists expected that more rapid PSA declines in response to primary ADT would be linked with extended survival. Conversely, reports suggest that these rapid responses to treatment may be indicative of more aggressive disease [59]. Even though ADT is advantageous in most patients exhibiting BCR, there are men whose disease will still progress despite treatment. When this occurs in the absence of any metastatic disease, it is known as non-metastatic castrate-resistant prostate cancer (nmCRPC). Castrate-resistant disease is often distinguished by two successive PSA increases when testosterone levels are <0.5 ng/mL [60]. 

Whilst the majority of men diagnosed with localised PC may be cured, their risk of treatment failure and death from subsequent metastatic disease increases significantly with their risk grouping at diagnosis (for example, at least 50% of all high-risk patients will not be cured). ADT is the standard initial treatment for those patients that develop distant metastases [61]. Although sequential PSA measurements can be used to assess response to ADT, validated definitions of disease progression or response to treatment with regard to PSA levels have yet to be established for this scenario. However, studies have demonstrated that advanced PC patients with a PSA measurement of <4 ng/mL after around 7 months of ADT have an improved outcome compared to patients with PSA levels > 4 ng/mL [62]. Additional studies have similarly shown that lower PSA measurements after ADT lead to better outcomes [63]. As is the case with localised disease, resistance to ADT also occurs in the metastatic setting, leading to the formation of metastatic castrate-resistant prostate cancer (mCRPC). PSA can be utilised to evaluate the response of mCRPC to treatment [64,65,66,67].

## 3. Techniques to Improve the Diagnostic Accuracy of PC

As previously discussed, over-diagnosis and over-treatment are two well-documented issues of the use of PSA for screening and monitoring programmes. Richard Albin, who is credited with the discovery of PSA [68], published “The Great Prostate Hoax” in 2014, where he discusses how he never intended for his discovery to be used in a PC screening program, highlighting its two major limitations: (i) it is not cancer-specific and (ii) it cannot differentiate between slow growing and aggressive cancers. The low specificity of PSA for detecting disease can lead to a considerable number of men undergoing unnecessary biopsies in order to exclude or verify the presence of malignancy. This situation arises largely because various non-cancerous processes such as trauma, prostatitis and benign prostatic hyperplasia (BPH) can lead to increased serum PSA levels [17]. BPH is a significant confounding factor for PC diagnosis as the occurrence of this condition increases with age, with a prevalence of 8%, 50% and 80% reported in men in their 30s, 50s and 80s, respectively [69]. The number of false positive PSA-based diagnoses will of course depend on the threshold used. In the ERSCP trial a PSA threshold of ≥3.0 ng/mL was used to determine if a biopsy was required; approximately 75% of men who presented with PSA levels ≥ 3.0 ng/mL were confirmed as PC negative following a biopsy procedure [31]. PSA screening can also suffer from false negative results; it has been estimated that a cut off of 4.0 ng/mL will miss around 15% of PC cases, of which around 15% will have advanced Gleason scores [70], and that a cut off value of 4.1 ng/mL will only detect ~20% of PC cases [71]. In an attempt to overcome these limitations, studies have investigated the use of various PSA parameters/dynamics, along with the use of additional or adjunct tests, to improve the diagnostic specificity and prognostic potential of PSA (Table 2).

### 3.1. PSA Density

PCs can produce increased levels of PSA per volume of tissue compared to benign prostatic conditions. To take into account prostate volume, PSA density (PSAD) was introduced in the early 1990s by Benson et al. This was done in an attempt to improve the accuracy of serum PSA testing to distinguish between small-volume organ-confined PC and BPH [72]. PSAD is calculated by dividing serum PSA by the volume of the prostate gland, measured by either transrectal ultrasound or magnetic resonance imaging. Studies have shown that PSAD has the potential to influence biopsy decisions by helping to identify men that harbour clinically significant PC [73,74,75,76], with PSAD becoming a better marker for predicting clinically significant PC as PSA levels increase [77]. Further work has exhibited the potential of PSAD to determine PC aggressiveness and predict the presence of adverse pathology in patients undergoing RP [78,79]. These results suggest that PSAD may play a role in risk stratification, which could be especially important when deciding which patients may be eligible for AS [79,80,81]. Overall, PSAD represents a simple, inexpensive tool that, if validated, has the potential to identify patients that may forego unnecessary biopsies. 

### 3.2. PSA Dynamics

Changes in PSA parameters including doubling time (PSADT, time required for PSA levels to double) and velocity (PSAV, the rate of PSA change/year) can provide additional information over the evaluation of total PSA alone. Carter et al. introduced the concept of PSAV in 1992, performing multiple PSA measurements on serum samples obtained from men between 7–25 years prior to histological diagnosis or exclusion of PC; they found that while absolute PSA levels did not significantly differ between men with BPH and PC, the rate of change of PSA was significantly greater in those subjects with PC. They concluded that PSAV may act as an early biomarker for the development of PC [82]. Since this initial study, there has been some debate on the value of PSAV for diagnosing PC or providing a prognosis for PC in patients under AS [83,84]. However, there are studies that indicate that PSAV has potential as a prognostic/predictive biomarker in patients treated with RP [85,86,87,88] and RT [89,90]. The evidence thus far indicates that PSAV has better value in the post-treatment setting rather than in the pre-treatment setting. PSADT has shown promise as a predictive biomarker for PC detection on repeat biopsy, thus exhibiting the potential it has in the avoidance of unnecessary biopsies [91]. Studies have also assessed the clinical significance of PSADT before definitive therapy; here patients that exhibit longer pre-operative doubling times have been shown to have a better prognosis following treatment [92]. PSADT can additionally be used to monitor PC recurrence/progression following curative therapy [53,93], with a doubling time of <3 months associated with reduced survival times [93]. More recent work has demonstrated that PSADT can predict the occurrence of metastasis [94,95,96]. Although measuring PSAV and PSADT can provide additional information over the evaluation of total PSA alone, to date there is a lack of clear evidence to endorse the sole use of PSA dynamics in the clinic. Further prospective studies comparing the analysis of PSAV, PSADT and PSA are required [97]. 

### 3.3. Molecular Forms of PSA

PSA can exist in multiple forms within the blood. PSA found in serum can be classified as either free PSA (fPSA) or complexed PSA (cPSA). Whereas fPSA is unbound to carrier molecules/proteins, cPSA is bound to protease inhibitors (α1-antichymotrypsin, α2 macroglobulin or α1-antitrypsin) [98]. Assays that can measure these molecular forms can provide additional information over the assessment of total PSA levels [99,100,101]. fPSA levels are generally expressed as a percentage of total PSA (%fPSA). In general, men with PC have decreased levels of %fPSA when compared against men without PC [34]. As fPSA levels tend to decrease with PC, it can distinguish PC from BPH [99]. Unfortunately, there are limitations to the assessment of fPSA; this free form is less stable than complexed PSA in the blood, meaning sample processing has to be done soon after collection [102]. Additionally, DRE and biopsy procedures lead to a rise in the amount of fPSA in the blood [103]. Increasing prostate volumes have also been shown to lead to increased %fPSA values; as such, %fPSA is thought to only provide reliable data in patients whose prostate volume is <40 cm^3^ [104].

Studies have suggested that the measurement of fPSA can be most beneficial in patients whose PSA levels are between 4–10 ng/mL, with some reporting that the use of fPSA can provide a diagnostic sensitivity of 95% and a specificity of 93%; however, others have reported poorer corresponding values of 75% and 32% [105]. A meta-analysis carried out to assess the accuracy of measuring %fPSA for the diagnosis of PC in men with PSA concentrations ranging from 4–10 ng/mL demonstrated that this test had low sensitivity and specificity. The authors concluded that %fPSA is neither sensitive nor specific enough to be utilised by itself, and the results of these tests need to be combined with additional diagnostic methods in helping to inform whether a prostatic biopsy is required [105]. Oto et al. recently explored the potential of %fPSA when merged with other factors, demonstrating that the combination of %fPSA with total PSA and age in a predictive model increased the diagnostic potential of total PSA [106]. 

To circumvent some of the issues encountered with %fPSA, studies have investigated the use of molecular forms of PSA in diagnostic assays, including intact PSA (iPSA) and [2]proPSA [107]. The Prostate Health Index (PHI) assay, the 4-kallikrein panel (4Kscore) and the Stockholm-3 (STHLM3) model are each multiplex tests that incorporate various molecular forms of PSA. Each of these assays are detailed in the subsequent sections. 

### 3.4. Prostate Health Index

The PHI assay was developed to aid the detection of clinically significant PC. It is a score derived from total PSA, fPSA and [2]proPSA values using the formula ([2]proPSA/fPSA) × √total PSA [108]. [2]proPSA is a peptide precursor to mature PSA that is preferentially produced in malignant cells [109]. The perceived advantage of this test is that it allows clinicians to evaluate individual PSA parameters in combination with the overall score produced. The chief use of PHI within the clinic is to lower the number of unnecessary biopsies acquired from patients with PSA levels that are considered borderline, without losing the detection of aggressive tumours. 

The PHI test was approved in 2012 by the FDA for use in patients over 50, with PSA readings between 4–10 ng/mL and a negative DRE. Studies have shown that PHI is superior to %fPSA and total PSA in the detection of PC [110,111,112,113,114,115,116]. This greater accuracy in the detection of PC was particularly apparent in patients with PSA levels between 2–10 ng/mL [113]. PHI has also shown increased predictive accuracy for clinically significant/aggressive disease when compared against %fPSA and total PSA [116,117,118,119,120]. Between 15–45% of unnecessary biopsies can be avoided using the PHI test, depending on the cut-off values used [121]. The capacity of PHI-density (determined by dividing the PHI score by the prostate volume) to distinguish clinically significant PC has also been shown [122,123]. The combination of PHI with multi-parametric magnetic resonance imaging (mpMRI) has also been assessed, with PHI helping to determine the need for re-biopsy and improving the detection of clinically significant PC [123,124]. 

The PHI score has been demonstrated to impact patient management in the clinic, leading to biopsy deferrals when the patient PHI score was low and the decision to carry out a biopsy when the PHI score suggested that there was an intermediate/high probability of PC being present [125]. From a health-economic perspective, the cost-effectiveness of including PHI in the decision-making process for whether a prostatic biopsy is required has also recently been demonstrated [126,127]. As well as lowering the number of unnecessary biopsies, the prediction of BCR following RP is another potential use for the PHI test [128,129]. There are, however, some difficulties associated with the use of this test in the clinic. While it has been shown that PHI is an effective tool for risk stratification in both Asian and European populations, reports indicate that differing PHI reference ranges should be employed for distinct ethnic groups [130]. Like fPSA, studies have demonstrated that [2]proPSA also has some issues with molecular instability [131]. 

### 3.5. Four-Kallikrein Panel

Human kallikrein 2 (hK2) is a serine protease that shares 80% sequence homology with that of PSA. Studies have indicated that hK2 may have a role in distinguishing between patients with PC and those without malignant disease, while also having the ability to predict stage, grade and BCR in those patients treated with RP [132]. Using serum samples from the ERSPC trial, a prediction model was produced based on a panel of 4 kallikrein markers: total, free and iPSA in combination with hK2 levels. Commercialised by Opko Diagnostics, the 4-kallikrein panel (4Kscore), in conjunction with patient clinical data (age, DRE and previous biopsy results), generates a risk of the presence of high-grade PC. This model led to a better discrimination of high-grade PC when compared against total PSA and clinical variables alone [133,134,135,136]. 

Like %fPSA and PHI, the primary aim of the 4Kscore is to reduce disease over-detection by helping clinicians decide which patients require a biopsy. Its use is currently recommended in men undergoing either an initial or a repeat biopsy. The results from a large, prospective multi-institutional trial showed that the 4Kscore distinguished patients that had a Gleason score ≥7 from those that scored <7. Using a 6% cut-off value, the authors suggested that 30% of biopsies could be avoided whilst delaying a diagnosis of high-grade PC in only 1.3% of patients [137]. Further studies have demonstrated the potential of the 4Kscore to predict the presence of clinically significant PC [138,139,140,141,142,143]. As with PHI, the 4Kscore test has also been assessed when used in combination with mpMRI, with results showing that the 4Kscore improved the prediction of high-grade PC when utilised alongside mpMRI [144]. The ability of the 4Kscore to identify the presence of aggressive cancers across multi-ethnic populations has also recently been exhibited, thus demonstrating its wide clinical applicability [145]. 

Studies have established that use of the 4Kscore has the potential to significantly influence clinician and patient decision-making processes, leading to a reduction in the number of biopsies performed, while also increasing the likelihood of identifying aggressive PC [146]. The capacity of the 4Kscore to significantly reduce costs while also enhancing the quality of patient care has also been shown [147,148]. Other studies have investigated the 4Kscore for its ability to predict distant metastasis; 4Kscores from patients assessed at 50 and 60 years of age can stratify men into two cohorts in terms of their risk of developing metastatic disease 20 years following diagnosis [149]. 

### 3.6. The STHLM3 Model

Genome-wide association studies have produced convincing evidence for a genetic predisposition for PC in some patients. Single nucleotide polymorphisms (SNPs) have been described which account for around 30% of the hereditary risk for PC, offering novel areas for exploration into the pathogenesis of this disease [150]. The combination of a genetic score centred on these SNPs with PSA to improve the specificity of PSA testing alone has been investigated [151,152]. STHLM3 is a risk-based model for PC screening that combines 232 SNPs, a combination of plasma protein biomarkers (PSA, iPSA, fPSA, hK2, beta-microseminoprotein and macrophage inhibitory cytokine 1) and clinical variables (family history, age, prostate exam and previous biopsies) [153]. Studies have found that this model performed better than PSA alone for the detection of high-risk PC, exhibiting its potential to improve PC diagnosis by significantly reducing the number of unnecessary biopsies taken, while also preserving the same sensitivity to diagnose clinically significant PC [153,154,155,156,157]. 

### 3.7. PSA Glycosylation

Glycans are saccharides that can be bound to lipids, proteins and other glycans through glycosylation. Glycosylation is thought to be the most frequent post-translational modification and is essential to nearly all biological processes that occur in the body [158]. Aberrant glycosylation is a widespread characteristic within cancer cells that has been identified in most cancer types, and is often referred to as a “hallmark of cancer” [159]. A SNP that has an effect on PSA glycosylation has recently been linked to PC risk [160]. Developments in mass spectrometry technology have led to further research into glycan structures on tumour-associated proteins; differing studies have assessed whether a glycan signature on PSA may be utilised to improve its clinical efficacy [161]. The extent to which a protein/lipid is glycosylated is dependent on the expression of specific glycosylation enzymes in a cell, as well as the quantity of glycosylation sites present [162]; PSA contains a single N-glycosylation site [161]. Variations in PSA glycosylation states have been shown to occur in both PC cell lines [163] and in blood samples from patients with and without PC [164]. So far, around 50 PSA glycoforms have been defined, with some of these found to be present in aggressive PC. In particular, α2–3-linked sialic acid alterations to PSA in clinical samples have gained the most interest from researchers. The ability of α2,3-sialylated PSA to diagnose PC has been reported [165], with further studies also demonstrating its potential to differentiate high-risk PC from low- and intermediate-risk PC and BPH patients [166,167].

### 3.8. DNA Methylation

Epigenetic processes can affect the expression of genes, leading to alterations in malignancy-associated phenotypes including angiogenesis, growth, invasion and migration. Numerous alterations in DNA methylation have been distinguished between cancerous and benign prostate tissues [168]. As a result, aberrant DNA methylation is an epigenetic change that has promise as a diagnostic or prognostic PC biomarker [169]. The Epigenetic Cancer of the Prostate Test in Urine (epiCaPture) is a DNA methylation urine test for high-risk PC. It is designed to measure DNA hypermethylation within the regulatory regions of six PC-associated genes (*GSTP1*, *SFRP2*, *IGFBP3*, *IGFBP7*, *APC* and *PTGS2*) [170]. Increased methylation levels within epiCaPture genes have been shown to be associated with higher PC aggressiveness. The authors concluded that epiCaPture could be used as an adjunct to PSA, aiding in the selection of patients that should undergo a prostatic biopsy [170].

## 4. Alternative Diagnostic Biomarkers to PSA

There are biomarkers other than PSA that have a role in the pre-diagnostic setting. The ideal biomarker here should have the ability to increase the likelihood of identifying clinically significant PC on biopsy tissues, while also leading to the avoidance of biopsies in men who do not require one due to the absence of clinically significant PC. These types of biomarkers can be categorised into those employed to decide who to biopsy (SelectMDX, TMPRSS2-ERG score and the miR Sentinel test) and those utilised to choose when to re-biopsy (ConfirmMDx, prostate cancer antigen 3 [PCA3] and the Prostate Core Mitomic Test).

### 4.1. SelectMDx

The SelectMDx assay is a urine-based test designed to give the probability of detecting PC after a biopsy, in addition to the likelihood of low-grade versus high-grade disease. SelectMDx is performed after prostatic massage, with mRNA levels of *DLX1* and *HOXC6* genes (reported to be good predictors for the detection of high-grade PC [171,172]) measured within the urine through qRT-PCR. *DLX1* and *HOXC6* gene expression levels are then combined with clinical parameters (PSA density, age, DRE and family history information). Van Neste et al. postulated that the use of this test could lead to a 42% decrease in the total number of biopsies carried out, with a 53% reduction in the number of unnecessary biopsies [172]. Further studies have shown that this test can help clinicians identify men at risk of clinically significant PC, thus aiding the initial biopsy decisions and helping to reduce the number of unnecessary biopsies [173,174,175]. Analyses have indicated that the use of SelectMDx before proceeding to biopsy could lead to an increase in quality-adjusted life years (a measure of disease burden that takes into account both the quantity and quality of life lived) while also saving healthcare costs [176,177,178]. The SelectMDx test was included in the 2020 National Comprehensive Cancer Network (NCCN) guidelines for the early detection of PC. While there have been reports indicating that SelectMDx outperforms other tests such as PHI in screening for the presence of high-grade PC before biopsy [179], more recent papers have led to questions over the worth of the SelectMDx assay [180,181].

### 4.2. TMPRSS2-ERG Score

Chromosomal translocations are a common occurrence in cancer [182]. Tomlins et al. identified candidate oncogenic genomic rearrangements based on outlier gene expression; through this method, they discovered chromosomal translocations that lead to the fusion of the androgen-regulated gene transmembrane protease serine 2 (*TMPRSS2*) and ETS transcription factors (predominantly ETS-regulated gene [*ERG*]), also known as TMPRSS2-ERG [183]. Experiments indicated that the androgen-responsive promoter of *TMPRSS2* facilitated the overexpression of *ERG* in PC [183]. This chromosomal rearrangement has been identified in pre-cancerous prostatic conditions (e.g., intraepithelial neoplasia) and has been shown to be specific to PC [183,184,185,186,187]. TMPRSS2-ERG gene fusions occur in ~50% of PCs [186,188,189]; in those cases that overexpress ERG, up to 90% will be positive for the gene fusion [183,190,191,192]. 

Similar to the SelectMDx test, qRT-PCR can also be used to measure TMPRSS2-ERG mRNA in urine samples following prostatic massage. Simultaneous assessment of PSA mRNA allows a TMPRSS2-ERG score to be generated from the TMPRSS2-ERG mRNA/PSA mRNA ratio. Studies have illustrated that the assessment of TMPRSS2-ERG gene fusions in urine has the potential to predict the diagnosis of PC from subsequent prostatic biopsy samples [188,193]. Others have shown a correlation between TMPRSS2-ERG gene fusion, grade [194,195] and stage [196] at diagnosis, with analysis of the gene fusion also demonstrated to have the ability to predict the risk of clinically relevant PC after a prostatic biopsy [189]. Studies have additionally investigated whether TMPRSS2-ERG gene fusions can be utilised to assess PC aggressiveness in patients undergoing AS, thereby having use as a prognostic biomarker when assessed in prostatic tissues samples [190].

### 4.3. miR Sentinel Test

Exosomes and prostate-specific exosomes (prostatosomes) are small (30–150 nm) double lipid membrane-bound extracellular vesicles that are generated within cells through internal budding of multi-vesicular body membranes. For endosomal contents to be released from cells, they require endocytosis and fusion of their membranes with the cellular plasma membrane. The contents of prostatosomes can be released into urine, semen and blood, with these prostatosomes containing various molecules including proteins, lipids and nucleic acids [197]. These substances not only play key roles in cellular signalling, but have also been shown to be regulators of tumourigenesis and cancer progression, including immune suppression, angiogenesis, cell migration and invasion [198]. As such, prostatosomes are a rich source of biomarkers for PC diagnosis and prognosis. In comparison to men without disease, PC patients have increased numbers of serum-detected exosomes, with reports indicating that these higher levels may also correlate with higher Gleason scores [199]. Prostatosomal contents including PSA and TMPRSS2-ERG have also been detected within urine-derived exosomes from PC patients [200]. 

The miR Sentinel test is a recently developed platform that analyses small non-coding RNAs (sncRNAs) acquired from urinary exosomes [201]. This platform consists of three different tests; the Sentinel PCa test (distinguishes patients with PC from those in which there is no evidence of PC), the miR Sentinel CS test (differentiates patients that have PC into those with low-risk disease and those with intermediate/high-risk PC) and the miR Sentinel GH test (classifies patients with PC into those with low- and favourable intermediate-risk disease and those patients with high-risk PC). Each of the tests demonstrated sensitivities and specificities above 90%, highlighting their potential to diagnose and classify PC in a non-invasive manner with great precision [201]. Further validation of these tests is required in other independent patient cohorts and racially diverse patient groups. 

### 4.4. ConfirmMDx

ConfirmMDx (MDxHealth, Inc) is an assay based upon DNA methylation and is designed to separate patients that have PC from those with a true negative biopsy result. The methylation status of Glutathione S-Transferase Pi 1 (*GSTP1*), Ras association (RalGDS/AF-6) domain family member 2 (*RASSF2*) and Adenomatous Polyposis Coli (*APC*) are evaluated using this assay [202]. The assay requires a minimum of eight core biopsy specimens obtained from specific prostatic regions. The advantage of using this assay is that molecular DNA alterations in prostatic cells that are adjacent to PC lesions, which would otherwise be diagnosed as histologically benign, can be identified. This is a result of the “halo effect” that the tumour has on surrounding normal tissues [203]. A positive ConfirmMDx result in biopsy tissue that has been labelled as cancer negative by a pathologist indicates that tumour cells were missed in the biopsy procedure. Thus far, its use has been validated in two different studies [202,204], exhibiting the potential ConfirmMDx has in helping to decrease the number of unnecessary repeat biopsies. In those patients that produce positive results, DNA methylation intensities also aid in the identification of men with high-grade disease [205]. While previous work was predominantly carried out in Caucasian men, recent work has demonstrated that this test is also effective in African American patients [206]. The Prostate Assay Specific Clinical Utility at Launch (PASCUAL) study (NCT02250313) is currently underway, examining the clinical value of the ConfirmMDx test in urologic practices within the US.

### 4.5. PCA3

The prostate-specific *PCA3* gene encodes a non-coding RNA that exhibits up to a 66-fold upregulation in prostatic tumours, with studies showing it to be present in >90% of PC cases [207,208,209]. In light of encouragingly high sensitivity and specificity results from tissues, numerous studies investigated the assessment of PCA3 levels non-invasively using urine [209,210,211]. Through qRT-PCR, PCA3 mRNA can be readily measured in urine samples following prostate massage. A PCA3 score is calculated from the PCA3 mRNA/PSA mRNA ratio, multiplied by 1000. Analysis of PSA mRNA levels, as performed in the TMPRSS2-ERG score assay, is required to control for the quantity of prostate epithelial cells in the urine. A score below the cut-off of 25 is interpreted as a negative result (there is a decreased likelihood of PC being present), with scores ≥25 indicating an increased probability that PC is present. However, there is debate over what PCA3 cut-off score should be used [212,213]. 

The PCA3 Progensa test was approved by the FDA in 2012 for use in suspect PC cases with equivocal PSA/DRE/biopsy results. Studies have demonstrated that PCA3 has an acceptable diagnostic accuracy and can help guide decisions on whether or not to carry out an initial biopsy, thus reducing the number of unnecessary biopsies [214]. The addition of PCA3 scores to individual risk estimation models, which included clinical factors, age and patient race, has been shown to improve PC stratification [215]. Wei et al. concluded that PCA3 measurement can reduce the under-detection of high-grade disease in initial prostatic biopsies, while also minimising the over-detection of low-grade PC in repeat biopsies [215]. Other studies have also demonstrated that PCA3 can supplement PSA and other clinical information to help give a more accurate prediction of the outcome from repeat biopsies [216,217].

As with the previously discussed biomarkers, the combination of PCA3 score with mpMRI has also been examined. The PCA3 score in men with a suspicious area for PC after mpMRI was higher than that of patients with no suspicious regions post-mpMRI; these results indicated that the PCA3 test could be used to pick those patients that should be referred for an mpMRI scan [218]. The addition of the PCA3 score to mpMRI was also shown to improve the predictive accuracy of mpMRI [219,220]. New methods for PCA3 detection are under development to enable PCA3 tests to be carried out in developing countries and to allow the assay to be used as a point-of-care test [221,222,223,224].

Studies have indicated that PCA3 could be employed to influence decisions between AS and more radical treatment options. It has been suggested that a threshold score of 20 could be used to identify men with clinically insignificant PC who would be eligible for AS, while a threshold of 50 could identify men at higher risk of having clinically significant PC who are good candidates for radical therapy [213]. However, the correlation of PCA3 score and PC aggressiveness is under debate, with some studies exhibiting a relationship between PCA3 score and Gleason score [225,226,227,228,229], whilst other do not [230,231]. Additionally, comparative studies indicate that PHI outperforms PCA3; PHI exhibited increased accuracy for PC prediction in initial and repeat biopsies [232], with PHI also superior in the detection of aggressive disease [233]. While it is improbable that PCA3 will replace PSA as the frontline biomarker for PC, the measurement of both PCA3 and PSA could lead to greater specificity for PC diagnosis.

### 4.6. Combined PCA3 and TMPRSS2-ERG Tests

Considering the significant heterogeneity seen within PCs, and the fact that not all PCs will express PCA3 or possess TMPRSS2-ERG gene fusions, researchers have investigated the use of multiplexed assays using both PCA3 and TMPRSS2-ERG gene fusions to improve PC diagnosis [188,189]. The Mi-Prostate Score and ExoDx Prostate IntelliScore (EPI) test are examples of these assays. The Mi-Prostate Score uses PCA3 and TMPRSS2-ERG urine scores with serum PSA levels; this combination was shown to enhance the ability of serum PSA to predict PC [234,235]. The EPI assay is an exosome-based urine assay which does not require a prostatic massage. It assesses PCA3 and TMPRSS2-ERG mRNA levels, with the SAM pointed domain-containing Ets transcription factor analysed for RNA normalisation. The EPI assay has been suggested for use in men with increased PSA levels in order to give a risk assessment for the presence of clinically significant PC at the initial biopsy [236,237]. The EPI test also has the potential to rule out the presence of high-grade disease using repeat biopsy tissues [238]. Results from the EPI test have been shown to influence biopsy decision making within the clinic [239]. Trials to confirm the performance of the EPI assay in men presenting for initial (NCT04720599) and repeat (NCT04357717) biopsies are currently underway.

### 4.7. Prostate Core Mitomic Test

Various cumulative genetic and epigenetic alterations within a cell contribute to the process of cell transformation. Although some of these genetic changes lead to cancer formation, early genetic alterations can lead to the growth of pre-neoplastic daughter cells in a particular area of the tumour field. While changes in cellular morphology enable the transformed cells to be diagnosed through histopathology, a population of pre-neoplastic daughter cells may be present that would not be diagnosed using this method, illustrating the concept of field cancerisation [240]. In PC, molecular field characterisation has been described for gene expression profiles and genomic instability. One study demonstrated that a 3.4-kb mitochondrial genome deletion (3.4mt∆) had potential as a biomarker for PC detection using biopsy samples. As a result of field cancerisation, the levels of 3.4mt∆ in clinical samples from malignant biopsy specimens were similar to the levels that were acquired from samples close to the malignant tissue. The authors concluded that large-scale mitochondrial DNA deletions may have use in the diagnosis of PC through their ability to define benign, malignant and proximal to malignant tissue, thereby helping resolve false from true-negative results [241]. The utility of this 3.4mt∆ in identifying men who do not need a repeat biopsy has been shown [242]. The Mitomic Prostate Core Test was subsequently developed for use in existing negative prostate biopsy tissue to assess if PC was missed in the initial biopsy. Further studies have demonstrated the usefulness of this assay in addressing sampling error issues encountered with prostate needle biopsies, with the test contributing to the earlier detection of PC when clinicians included the test in their re-biopsy decision-making process [243]. 

## 5. Biomarkers That Can Predict PC Aggressiveness

Definitive treatment for PC can lead to significant complications. Biomarkers that give an indication of disease aggressiveness in patients who have already been diagnosed would help clinicians decide who should be considered for AS and who should undergo radical treatment. This would assist in the identification of patients who could benefit from treatment, while also reducing the treatment risks and economic costs for those who are unlikely to benefit. 

### 5.1. Oncotype DX Genomic Prostate Score Assay

Predictive gene expression signature assays have been developed to help identify cohorts of patients that gain specific benefits from certain therapies. Signatures of breast cancer RT and chemotherapy response [244,245], and also treatment-predictive signatures for lung cancer [246] are successful, clinically useful examples of these. The Oncotype Dx assay, developed by Genomic Health, is a commercial gene signature assay that has gained significant popularity for identifying cohorts of breast cancer patients that gain benefit from adjuvant chemotherapy [247]. As a result of successful studies in breast, the applicability of an adapted test to PC has been examined. The Oncotype DX Genomic Prostate Score assay is carried out on prostatic biopsy tissue. It was designed to aid treatment selection at the time of diagnosis in patients with low- or intermediate-risk disease, enabling both patients and clinicians to make more informed choices between AS and immediate radical treatment [248]. This test is based on the expression pattern of 12 genes that characterise four separate pathways known to be involved in PC development and progression (proliferation, cellular structure/organisation, stromal interactions and androgen signalling), along with five housekeeper genes. A final Genomic Prostate Score (GPS) ranging from 0–100 is calculated. This GPS can provide predictive information regarding the risk of identifying adverse pathology after RP (higher grade and stage disease) [248,249,250,251,252], aids in determining the risk of PC recurrence after surgery [250], and can also ascertain the risk of BCR and distant metastasis [250,252,253,254]. The cost-effectiveness of the GPS assay in directing treatment decisions (AS versus immediate treatment) has also been reported [255,256]. 

However, more recent work has highlighted some limitations of the Oncotype DX GPS. Lin et al. tested the value of the GPS in predicting the presence of higher-grade disease at surgery in low-risk PC patients who were treated with RP after initial surveillance. They found that GPS did not significantly improve the stratification of risk for adverse pathology over the measurement of PSAD and diagnostic Gleason Grade alone [257]. Another study showed that the histopathological features which are present in PC biopsies, but are not usually reported, correlated with the GPS score. The authors suggest that more comprehensive analysis of PC histopathology could be used as a substitute for some of the information obtained from this test [258].

### 5.2. Prolaris

The Prolaris assay, developed by Myriad Genetics, is a tissue-based test intended for use in patients with newly diagnosed localized low- or intermediate-risk PC. This test is designed to enable clinicians to better define a monitoring/treatment strategy for these patients, identifying those who can be directed safely to AS and those that would benefit from treatment intensification. It is based on the expression patterns of 31 genes involved in cell cycle progression (CCP), in addition to 15 housekeeper genes. Overexpression of the CCP genes suggests that the cancer cells are rapidly dividing, while decreased expression signifies slower growth and a less aggressive cancer [259]. The Prolaris score or CCP score is reported on a scale ranging from 0 to 10, where higher scores are indicative of a more aggressive tumour [260]. The CPP score has been shown to give significant pre-treatment prognostic information that can be used to help determine which patients can be managed conservatively [261,262], with additional studies demonstrating that this assay has the ability to provide prognostic information for men undergoing either RP [259,263,264,265,266] or RT [267]. Higher CCP scores have also been shown to be linked with a higher risk of systemic disease [268] and can predict metastasis after either RT or surgery [269]. Results from the Prolaris assay have influenced therapy decisions within the clinic; there has been an increase in the proportion of patients undergoing AS in those that have been classified as low-risk by the Prolaris test, and intensification of treatments in those whose test results indicted the presence of more aggressive cancer [270,271,272]. While the potential benefits of the Prolaris assay have been exhibited, its value is limited by the retrospective nature of many of the studies performed; largescale, prospective trials are needed [260]. Additionally, the cost-effectiveness of the Prolaris test is still under debate [273,274].

### 5.3. ProMark

The ProMark quantitative immunofluorescence test was developed in an attempt to give clinicians the ability to predict PC aggressiveness, irrespective of whether biopsy cores came from low- or high-grade tumour regions, therefore accounting for sampling variation and PC heterogeneity. In a study carried out by Shipitsin et al., tissue regions with the lowest and highest grades were isolated in prostatectomy samples from the same patients; a panel of protein biomarkers was identified that predicted PC aggressiveness and outcome from both low- and high-grade areas [275]. This test is based on the expression patterns of eight proteins (DERL1, CUL2, SMAD4, PDSS2, HSPA9, FUS, pS6 and YBOX1) with known functions related to proliferation, tumour-associated signalling pathways and stress response, altogether providing information about tumour aggressiveness from formalin-fixed, paraffin-embedded (FFPE) tissues [276]. The primary function of the ProMark test is to separate candidates for AS from those that require RP, in addition to ascertaining those patients with favourable/non-favourable pathology. Although not yet validated, the test has the potential to accurately stratify low- and high-risk PC patients using biopsy samples.

### 5.4. Decipher

The Decipher test, developed by GenomeDx, is a genomic signature that was developed to help identify aggressive PC and improve the prediction of early PC metastasis using information from the primary tumour after RP. This test analyses the RNA expression levels of 22 genes (involved in cellular differentiation, proliferation, cell cycle, motility, adhesion, immune modulation and androgen signalling) detected in the primary tumour and was developed by modelling differential RNA expression patterns in early metastatic tissues versus controls [277]. The final Decipher score ranges from 0–1, with higher scores (0.61–1) associated with a higher probability of metastasis. This genomic classifier has gained interest for its use in patients after RP and can predict both the 5- and 10-year metastatic risk [278,279,280]. A recent meta-analysis conducted by Spratt et al. showed that Decipher can improve the prognostication of patients post-RP; the 10-year cumulative metastatic incidence rates after RP were 5.5%, 15.0% and 26.7% for patients that were deemed low-, intermediate- and high-risk using the Decipher test [281]. These results are supported by another study showing that transcriptional profiles can stratify patients into cohorts, separating those who will develop metastasis after RP from those who will not [282]. A recent study highlighted how Decipher, in combination with standard clinicopathologic variables, can lead to better risk-stratification when combined with current guidelines [283]. While the test was developed from the analysis of primary tissue after RP, the ability of the Decipher test to predict metastasis using biopsy tumour tissue has also been shown [284,285]. 

The potential of Decipher to predict BCR after surgery has also been established [286]. Patients exhibiting BCR after RP often have varied outcomes and thus present a management dilemma to clinicians; initial studies showed the ability of the Decipher test to predict metastasis in these patients, exhibiting its potential to identify men who require earlier initiation of treatment after BCR [287]. More recent studies have demonstrated that Decipher can be used to predict the absence of adverse pathology in low- and intermediate-risk PC patients, with the authors suggesting that Decipher may have a role in predicting which newly diagnosed patients are good candidates for AS [288]. Furthermore, Decipher scores have been shown to have potential in determining those patients who are most suitable for RT following RP [289,290]. The ability of the Decipher test to alter clinical decisions regarding the use of adjuvant treatments has been reported [291,292]. Altogether, data from several studies has demonstrated the clinical usefulness of the Decipher test, exhibiting its potential to significantly improve the personalisation of PC treatment [293]. 

### 5.5. Ki67

Ki67 is a nuclear protein related to ribosomal RNA synthesis. This protein is used as a marker for tumour proliferation, with analysis of Ki67 levels typically carried out through immunohistochemistry on FFPE tissues. Staining is described as the percentage of Ki67-positive cells within the total number of cancer cells present. Ki67 has been shown to be a prognostic and predictive biomarker in breast cancer [294]. Within PC, a higher percentage of Ki67-positive cells seems to have prognostic value for BCR, distant metastasis and survival in patients treated with either surgery or RT [295,296,297,298]. A recent meta-analysis incorporating 21 studies, comprised of 5419 patients, demonstrated that after curative-intent treatments, high Ki67 expression was a poor prognostic factor for disease-specific survival, disease-free survival, rate of distant metastases and overall survival. The authors concluded that Ki67 should be integrated into the clinic for use in PC patients [299]. However, despite the fact that Ki67 is one of the best validated prognostic markers that has been in use for over 30 years, some maintain that this protein is not yet ready for use in the clinic. High levels of variability in scores have been observed between different cohorts of PC patients, with scores ranging from 2.1% to 28% [300]. This issue seems to be particularly relevant in high-risk patients, in whom significant inter- and intra-prostatic Ki-67 heterogeneity has been reported [301]. The cut-offs used to distinguish a negative from a positive score also differ greatly between studies; this lack of standardisation across pathology laboratories contributes to the limitations of Ki67 as a PC biomarker [302]. 

### 5.6. MicroRNAs

MicroRNAs (miRNAs) are single stranded, small non-coding RNA molecules (~20 nucleotides in length) that function as post-transcriptional gene regulators through their ability to bind to complementary base pairs within specific mRNAs [303]. Alterations in miRNA profiles have been identified in PC. It has been suggested that miRNAs can regulate PC stem cells, cellular proliferation and differentiation, thereby influencing disease development and progression [304,305]. Studies showing that miRNAs are present in human blood in a very stable form [306] led to the development of miRNA signatures from blood samples in an attempt to improve the accuracy of PC diagnosis and prognosis. One such study identified a panel of 14 miRNAs, known as the miR risk score, which was able to discriminate Gleason grade and predict BCR following RP [307]. A further study showed that miR-16, miR-195 and miR-148a expression was correlated with Gleason scores ≥8, and that these three miRNAs could stratify patients into intermediate- and high-risk Gleason scores [308]. Several PC studies have also investigated miRNA signatures from urine samples to differentiate healthy patients or those with BPH from those with PC [309,310]

### 5.7. Phosphatase and Tensin Homolog

Phosphatase and tensin homolog (*PTEN*) is a well characterised tumour suppressor gene involved in the regulation of the phosphatidylinositol 3-kinase (PI3K) pathway. Loss of function of PTEN and the resulting de-regulation of the PI3K pathway is regarded as one of the most common driver events in PC development [311]. Loss of PTEN function has been shown to occur in ~40% of PC cases, especially in those with TMPRSS2-ERG gene fusions [312]. Although immunohistochemistry is typically used to evaluate PTEN loss, fluorescence in situ hybridisation (FISH) can be utilised where ambiguous immunohistochemistry results have been obtained [313]. Several studies have examined the use of PTEN loss as a biomarker in PC; one study suggested that patients exhibiting PTEN loss in Gleason score 6 tumours, identified from biopsy tissue, were at higher risk of having their score upgraded using samples obtained at RP [314]. Other investigations have demonstrated that loss or even just a decrease in PTEN expression is correlated with higher Gleason scores, more advanced disease stage, metastasis, BCR and disease recurrence [315,316,317,318,319]. Furthermore, shorter survival times have been reported in advanced PC with PTEN loss when treated with abiraterone acetate [320]. Apart from the removal of the tumour suppressive function, PTEN loss has also been associated with AR signalling suppression and inhibition of androgenic genes [321]; this may drive PC into an androgen-independent phenotype, ultimately reducing the efficacy of ADT.

## 6. Predictive Biomarkers

Predictive biomarkers indicate the likelihood of a particular treatment providing a therapeutic benefit. These biomarkers can therefore be used to aid treatment selection, enabling the identification of patients that are most likely to gain benefit from a particular therapy, whilst sparing others from the side effects of ineffectual treatment. Here, we provide an overview of a selection of predictive biomarkers that are currently being researched.

### 6.1. Post-Operative Radiation Therapy Outcomes Score

Although RT post-RP can significantly improve clinical outcomes, recent work does not support the routine administration of adjuvant RT post-RP [322]. It has been suggested that certain patient cohorts are more likely to gain benefit from its use; identification of these patients will improve their outcome while sparing the risk of developing radiation-induced side effects in those unlikely to gain a clinical benefit. Unfortunately, as of yet no gene signature has been clinically validated to predict RT response in PC patients. To begin to address this clinical issue, one study has developed and initially validated a 24 gene signature to predict RT response. This Post-Operative Radiation Therapy Outcomes Score (PORTOS) was developed using gene expression data from prostatic adenocarcinomas in patients who received a RP with or without adjuvant RT. Results demonstrated that the distant metastatic rate at 10 years for patients with a high PORTOS who received RT was lower than that observed for patients with a high PORTOS who did not receive RT (4% vs. 35%). While the authors suggested that PORTOS could be used to predict outcomes post-RT, thereby identifying which patient cohort should receive RT, they also demonstrated that other prognostic tools such as Decipher and the CCP score did not predict RT response [323]. 

### 6.2. DNA Repair Defects

Both pre-clinical and clinical reports indicate that DNA damage response pathways have a significant part to play in the progression of PC [324]. DNA repair defects are thought to be relatively frequent in more advanced PC, with genetic abnormalities that inhibit DNA repair shown to be present in mCRPC tumours [325]. It is thought that the identification of alterations in DNA repair pathways may be predictive of response to certain therapies. Poly (adenosine diphosphate [ADP]–ribose) polymerase (PARP) has a part to play in numerous aspects of DNA repair. PARP inhibitors are a class of anti-cancer agents that work through inducing synthetic lethality; this is a process where the PARP inhibitor, in combination with either an inherent genetic defect or another therapy (such as RT), cause irreparable DNA damage and cell death [326]. PARP inhibitors initially demonstrated their potential as an anti-cancer therapy in patients with BRCA1/2 mutations and they have become a standard treatment for patients suffering from ovarian and breast cancer. Olaparib and rucaparib are PARP inhibitors that have been approved by the FDA for the treatment of mCRPC [326]. The identification of DNA repair defects in mCRPC patients has been shown to predict response to PARP inhibitors; however, not all DNA repair defects have the same impact on the efficacy of treatment [327]. While the majority of data for PARP inhibitors has been generated for mCRPC patients, there will be interest among the scientific and clinical communities on the results of studies concentrating on earlier disease stages.

### 6.3. Androgen Receptor

The androgen receptor (AR) is a nuclear hormone receptor transcription factor that plays a significant role in the function of prostatic cells through its ability to bind sex steroids and control transcription of androgen-dependent genes [328]. ADT is a common treatment for PC; however, although nearly all PCs respond to this treatment in the beginning, tumour recurrence and progression into castrate-resistant prostate cancer (CRPC) typically occurs [329]. While the progression of androgen-dependent PC to CRPC likely involves various mechanisms, AR and its signalling have been shown to play important roles in disease development, including the acquisition of acquired resistance to various ADT drugs [330]. Within CRPC, AR alterations have been shown to occur through overexpression of wild-type or constitutively active variants (AR-Vs), gene amplification and mutations [331]. AR-Vs, generated from alternative splicing or gene rearrangements, have the ability to regulate transcription. Although these AR-Vs are truncated proteins that lack the AR ligand-binding domain, they still have functional DNA-binding and transcriptional activation domains, resulting in ligand-independent constitutive activation that is not constrained by anti-androgen treatment [331]. The AR-V7 form is frequently detected in mCRPC and has gained clinical interest for its use as a biomarker to help select the most appropriate treatments [332]. A crucial decision in mCRPC management is when to administer an AR signalling inhibitor or a taxane; studies have shown that AR-V7 expression is associated with the resistance of mCRPC to enzalutamide and abiraterone [333,334,335], while its expression also appears to correlate with increased response to taxane chemotherapies [336]. AR-V7 in CRPC patients can be detected within both prostatic tissue samples and circulating tumours cells (CTCs) [332,337]; however, conflicting findings have been observed between CTC AR-V7 results and AR-V7 protein expression in biopsy samples acquired from the same patient [338]. The OncotypeDX AR-V7 Nucleus Detect (Epic Sciences) and the AdnaTest AR-V7 assay (Qiagen) have been developed for the assessment of the constitutively active AR variant in CTCs.

### 6.4. Immune Checkpoint Inhibition

Monoclonal antibodies targeting immune checkpoint inhibitors are being considered as a new therapeutic strategy for the treatment of mCRPC. Immune checkpoint inhibitors (cytotoxic T lymphocyte-associated protein-4 (CTLA-4) receptor and programmed death-1 (PD-1) receptor) are present on T lymphocytes; these receptors act as negative regulators of the immune response, setting a balance between an effective immune response (including the response of the immune system to cancer cells) and tolerance to antigens produced by normal cells of the body [339]. The over-expression of ligands for these receptors on cancer cells (leading to the activation of immune checkpoint inhibitors and the inactivation of immune cells) has been observed in PC, contributing to the escape of these cancer cells from the host’s immune response [339]. The concept that the CTLA-4 and PD-1 receptors might be utilised by cancer cells to avoid the immune system led to the development of monoclonal antibodies that could inhibit these receptors, with the hope that targeting them would lead to a more effective anti-tumour response from T lymphocytes. Although some studies have demonstrated that Ipilimumab (an anti-CTLA-4 monoclonal antibody) and Nivolumab (an anti-PD-1 monoclonal antibody) are effective treatments for advanced PC [340,341], others have shown mixed results from the use of these agents [339]. It is thought that only certain patients are eligible for immunotherapy: those presenting with either high expression levels of CTLA-4 and PD-1 receptor ligands on cancer/stromal cells, or increased amounts of the immune checkpoint inhibitor receptors on immune cells. As such, it is believed that these proteins may act as biomarkers that could predict/monitor immunotherapy effectiveness [342,343]. 

Research into the predictive potential of genomic biomarkers for immunotherapy is also ongoing. Tumour mutational burden (TMB) can be used to describe the number of mutations in a tumour cell. Patients suffering from advanced PC have been shown to exhibit higher levels of TMB [344,345]. While the prediction of PC patient reaction to immunotherapy is complex, increased levels of TMB have been linked to better response [346]. It is believed that a higher TMB causes the production of increased levels of neoantigens (mutated antigens that are only expressed by cancer cells), which leads to a higher probability of an effective T-cell-dependent anti-cancer response [347]. Additional genomic predictive biomarkers for response to immunotherapy have also recently been identified; mutations within cyclin-dependent kinase 12 (CDK12), a tumour suppressor protein with roles connected to genomic stability [348], have also been demonstrated to lead to the creation of neoantigens [349]. It is thought that CDK12-altered PCs may respond favourably to immune checkpoint inhibitors [350]. 

## 7. Limitations and Future Perspectives of PC Biomarker Assays

The function of the prostate is to perform as a secretory gland, secreting proteins including PSA into seminal fluid. As such, liquid-based biomarkers, such as those acquired from the blood or urine, are well placed to act as PC-specific biomarkers. The identification of biomarkers in liquid biopsies has significant advantages over tissue-based techniques as they can be obtained easily in a less invasive manner. Liquid biopsies can also be routinely taken pre-, post- or on-treatment, meaning continual patient monitoring can be achieved, while tissue biopsies give only a limited snapshot of the tumour. Tumour heterogeneity is a significant problem for tissue-based biopsy tests, as results can only be determined from the area that the tissue samples are acquired from [351,352]. Liquid biopsies, in comparison, have the potential to give a comprehensive view of both primary and metastatic cancers. Urine samples in particular have specific advantages in PC management; as a result of the proximity of the bladder to the prostate, urine can contain biomarkers that reflect PC development and progression. 

Of the liquid-based assays, PSA is the best validated and most widely used biomarker employed by clinicians. This is likely to remain the case for the present, despite limitations associated with its use. To overcome some of these issues, studies have examined the use of different PSA parameters/dynamics. The combination of PSA with adjunct tests is also being studied in an attempt to enhance the diagnostic specificity and prognostic potential of PSA. Of the tissue-based biomarker tests discussed, Oncotype DX Prostate, ProMark, Decipher and Prolaris are the best validated thus far. While these tissue-based biomarker assays have the potential to influence the management of PC patients, there are a number of issues that are currently restricting their use: (i) Direct comparison of Oncotype DX, Prolaris and Decipher to one another has shown that prognostic outcomes can differ depending on the test used [353,354]. (ii) Many of these assays were developed and initially validated in cohorts of patients who were mostly white European or white American men, with limited initial research performed into the value of these tests in African American men, who are recognised as having poorer outcomes. While some of the assays have been validated and shown to provide benefit in diverse racial groups [250,355,356,357], racial differences across the gene expression panels used for PC prognosis have been identified [358]. (iii) Lastly, the clinical usefulness of these multigene signatures has yet to be prospectively validated in a randomised clinical trial. Regardless of these shortcomings, present NCCN recommendations assert that Prolaris, Decipher, ProMark and Oncotype DX Prostate can be used for risk stratification in patients with either low- or favourable intermediate-risk PC [359]. 

## 8. Conclusions

Significant advances continue to be made in the field of PC. Although the widespread use of PSA levels for PC diagnosis and management led to criticisms of over-diagnosing and over-treating patients, its use undoubtedly paved the way for investigations into more specific PC biomarkers. The biomarkers discussed in this review have the potential to contribute immensely to PC patient management by (i) cutting down on unnecessary biopsies, (ii) enhancing patient risk assessment and therefore treatment selection and (iii) leading to more selective treatments for PC patients with higher-risk disease. 

For any biomarker-based assay to become translated into the clinic and used routinely, studies need to demonstrate specificity, sensitivity and their potential to improve upon current clinical practices. That said, PC biomarker research holds much promise; linking novel PC-specific biomarkers with other techniques, such as clinical data, PSA levels, Gleason grading, disease staging and imaging would undoubtedly help improve the management of PC patients. Ultimately, we need implementation of many of the assays discussed into well designed randomised clinical trials in order to validate them; hopefully it is only a matter of time before this can be achieved. 

## Figures and Tables

**Table 1:** Overview of prostate cancer biomarker assays that are in development or have gained clinical approval. Pre-diagnostic biomarkers (blue), biomarkers used in biopsy-proven prostate cancer cases (yellow), predictive biomarkers (green). 3.4mt∆, 3.4-kb mitochondrial genome deletion; AR, androgen receptor; AR-V7, androgen receptor splice variant 7; BCR, biochemical recurrence; CTCs, circulating tumour cells; CTLA-4, cytotoxic T lymphocyte-associated protein-4; miRNA, microRNAs; PARP, poly (ADP-ribose) polymerase; PC, prostate cancer; PD-1, programmed death-1; PORTOS, Post-Operative Radiation Therapy Outcomes Score; PTEN, phosphatase and tensin homolog; RP, radical prostatectomy; RT, radiotherapy; sncRNAs, small non-coding RNAs; TMB, Tumour mutational burden.

Test	Analyte	Analyte Source	Outcome Provided by Test
SelectMDX	mRNA	Urine	Probability of detecting PC after prostatic biopsy, tumour grade
TMPRSS2-ERG score	mRNA	Urine	Probability of detecting PC after prostatic biopsy, tumour grade
miR Sentinel PCa	sncRNAs	Urine	Distinguishes patients with PC from subject with no evidence of PC, tumour grade
ConfirmMDx	Methylated DNA	Prostatic biopsy tissue	Separates patients that have PC from those with a true negative biopsy result, tumour grade
PCA3	mRNA	Urine	Probability of detecting PC after prostatic biopsy
Prostate Core Mitomic Test	3.4mt∆	Prostatic biopsy tissue	Resolves false from true-negative prostatic biopsy results
Oncotype DX Genomic Prostate Score	mRNA	Prostatic biopsy tissue	Tumour grade, BCR, metastasis, recurrence
Prolaris	mRNA	Prostatic biopsy tissue	Tumour aggressiveness, metastasis
ProMark	Protein	Prostatic biopsy tissue	Tumour aggressiveness
Decipher	mRNA	Primary tumour after RP, prostatic biopsy tissue	Tumour aggressiveness, BCR, metastasis
miR Sentinel CS/GH tests	sncRNAs	Urine	Tumour grade
Ki67	Protein	Primary tumour after RP, prostatic biopsy tissue	BCR, metastasis, survival
miR risk score	miRNAs	Serum	Gleason score, BCR
PTEN	Protein	Prostatic biopsy tissue	Gleason score, stage, metastasis, BCR, recurrence
PORTOS	mRNA	Primary tumour after RP	Predict RT response
DNA repair defects	mRNA	Prostatic biopsy tissue	Predict response to PARP inhibitors
AR-V7	Protein, mRNA	Prostatic biopsy tissue, CTCs	Predict resistance to AR signalling inhibitors or sensitivity to taxanes
CTLA-4 and PD-1 receptors, TMB	Protein, mRNA	Prostatic biopsy tissue, CTCs	Response to immunotherapy

**Table 2:** Overview of PSA-based diagnostic and prognostic assays. fPSA, free PSA; hK2, human kallikrein 2; iPSA, intact PSA; PHI, Prostate Health Index; PSA, prostate specific antigen; SNPs, single nucleotide polymorphisms.

Test	Fluid	Target
PSA density	Serum	PSA
PSA dynamics	Serum	PSA
%fPSA	Serum	PSA and fPSA
PHI	Serum	PSA, fPSA, [2]proPSA
4Kscore	Serum	PSA, fPSA, iPSA, hK2
STHLM3 model	Serum	PSA, fPSA, iPSA, hK2, beta-microseminoprotein, macrophage inhibitory cytokine 1, 232 SNPs
PSA glycosylation	Serum	α2,3-sialylated PSA
epiCaPture	Urine	PSA and methylated GSTP1, SFRP2, IGFBP3, IGFBP7, APC, PTGS2

## Data Availability

Not applicable.

## References

[1] Adams J. (1853). The case of scirrhous of the prostate gland with corresponding affliction of the lymphatic glands in the lumbar region and in the pelvis. Lancet.

[2] Sung H., Ferlay J., Siegel R.L., Laversanne M., Soerjomataram I., Jemal A., Bray F. (2021). Global Cancer Statistics 2020: GLOBOCAN Estimates of Incidence and Mortality Worldwide for 36 Cancers in 185 Countries. CA Cancer J. Clin..

[3] Siegel R.L., Miller K.D., Fuchs H.E., Jemal A. (2021). Cancer Statistics, 2021. CA Cancer J. Clin..

[4] (2020). NICE. https://www.nice.org.uk/Media/Default/About/what-we-do/Into-practice/measuring-uptake/prostate-cancer/nice-impact-prostate-cancer.pdf.

[5] Rawla P. (2019). Epidemiology of Prostate Cancer. World J. Oncol..

[6] Jahn J.L., Giovannucci E.L., Stampfer M.J. (2015). The high prevalence of undiagnosed prostate cancer at autopsy: Implications for epidemiology and treatment of prostate cancer in the Prostate-specific Antigen-era. Int. J. Cancer.

[7] Rodrigues G., Warde P., Pickles T., Crook J., Brundage M., Souhami L., Lukka H. (2012). Pre-treatment risk stratification of prostate cancer patients: A critical review. Can. Urol. Assoc. J..

[8] Chen N., Zhou Q. (2016). The evolving Gleason grading system. Chin. J. Cancer Res..

[9] Andreoiu M., Cheng L. (2010). Multifocal prostate cancer: Biologic, prognostic, and therapeutic implications. Hum. Pathol..

[10] Ruijter E.T., van de Kaa C.A., Schalken J.A., Debruyne F.M., Ruiter D.J. (1996). Histological grade heterogeneity in multifocal prostate cancer. Biological and clinical implications. J. Pathol..

[11] Loeb S., Vellekoop A., Ahmed H.U., Catto J., Emberton M., Nam R., Rosario D.J., Scattoni V., Lotan Y. (2013). Systematic Review of Complications of Prostate Biopsy. Eur. Urol..

[12] Litwin M.S., Tan H.J. (2017). The Diagnosis and Treatment of Prostate Cancer: A Review. JAMA.

[13] Mahal B.A., Butler S., Franco I., Spratt D.E., Rebbeck T.R., D’Amico A.V., Nguyen P.L. (2019). Use of Active Surveillance or Watchful Waiting for Low-Risk Prostate Cancer and Management Trends Across Risk Groups in the United States, 2010–2015. JAMA.

[14] Resnick M.J., Koyama T., Fan K.H., Albertsen P.C., Goodman M., Hamilton A.S., Hoffman R.M., Potosky A.L., Stanford J.L., Stroup A.M. (2013). Long-Term Functional Outcomes after Treatment for Localized Prostate Cancer. N. Engl. J. Med..

[15] van den Bergh R.C., Korfage I.J., Roobol M.J., Bangma C.H., de Koning H.J., Steyerberg E.W., Essink-Bot M.L. (2012). Sexual function with localized prostate cancer: Active surveillance vs radical therapy. BJU Int..

[16] Balk S.P., Ko Y.-J., Bubley G.J. (2003). Biology of prostate-specific antigen. J. Clin. Oncol..

[17] Lilja H., Ulmert D., Vickers A.J. (2008). Prostate-specific antigen and prostate cancer: Prediction, detection and monitoring. Nat. Rev. Cancer.

[18] Kan H.-C., Hou C.P., Lin Y.H., Tsui K.H., Chang P.L., Chen C.L. (2017). Prognosis of prostate cancer with initial prostate-specific antigen >1000 ng/mL at diagnosis. Onco Targets Ther..

[19] Stamey T.A., Yang N., Hay A.R., McNeal J.E., Freiha F.S., Redwine E. (1987). Prostate-specific antigen as a serum marker for adenocarcinoma of the prostate. N. Engl. J. Med..

[20] Catalona W.J., Smith D.S., Ratliff T.L., Dodds K.M., Coplen D.E., Yuan J.J., Petros J.A., Andriole G.L. (1991). Measurement of prostate-specific antigen in serum as a screening test for prostate cancer. N. Engl. J. Med..

[21] Parkes C., Wald N.J., Murphy P., George L., Watt H.C., Kirby R., Knekt P., Helzlsouer K.J., Tuomilehto J. (1995). Prospective observational study to assess value of prostate specific antigen as screening test for prostate cancer. BmJ.

[22] Lopez-Saez J.-B., Otero M., Senra-Varela A., Ojea A., MartÍn J.S., MuÑoz B.D., Fuentes J.V. (2004). Prospective Observational Study to Assess Value of Prostate Cancer Diagnostic Methods. J. Diagn. Med. Sonogr..

[23] Kouriefs C., Sahoyl M., Grange P., Muir G. (2009). Prostate specific antigen through the years. Arch. Ital. Urol. Androl..

[24] Potosky A.L., Feuer E.J., Levin D.L. (2001). Impact of screening on incidence and mortality of prostate cancer in the United States. Epidemiol. Rev..

[25] Draisma G., Etzioni R., Tsodikov A., Mariotto A., Wever E., Gulati R., Feuer E., De Koning H. (2009). Lead time and overdiagnosis in prostate-specific antigen screening: Importance of methods and context. J. Natl. Cancer Inst..

[26] Fenton J.J., Weyrich M.S., Durbin S., Liu Y., Bang H., Melnikow J. (2018). Prostate-specific antigen–based screening for prostate cancer: Evidence report and systematic review for the US Preventive Services Task Force. JAMA.

[27] Klotz L. (2013). Prostate cancer overdiagnosis and overtreatment. Curr. Opin. Endocrinol. Diabetes Obes..

[28] Grossman D.C., Curry S.J., Owens D.K., Bibbins-Domingo K., Caughey A.B., Davidson K.W., Doubeni C.A., Ebell M., Epling J.W., Kemper A.R. (2018). Screening for prostate cancer: US Preventive Services Task Force recommendation statement. JAMA.

[29] Sanda M.G., Dunn R.L., Michalski J., Sandler H.M., Northouse L., Hembroff L., Lin X., Greenfield T.K., Litwin M.S., Saigal C.S. (2008). Quality of life and satisfaction with outcome among prostate-cancer survivors. N. Engl. J. Med..

[30] Martin R.M., Donovan J.L., Turner E.L., Metcalfe C., Young G.J., Walsh E.I., Lane J.A., Noble S., Oliver S.E., Evans S. (2018). Effect of a low-intensity PSA-based screening intervention on prostate cancer mortality: The CAP randomized clinical trial. JAMA.

[31] Schröder F.H., Hugosson J., Roobol M.J., Tammela T.L., Zappa M., Nelen V., Kwiatkowski M., Lujan M., Määttänen L., Lilja H. (2014). Screening and prostate cancer mortality: Results of the European Randomised Study of Screening for Prostate Cancer (ERSPC) at 13 years of follow-up. Lancet.

[32] Schröder F.H., Hugosson J., Carlsson S., Tammela T., Määttänen L., Auvinen A., Kwiatkowski M., Recker F., Roobol M.J. (2012). Screening for prostate cancer decreases the risk of developing metastatic disease: Findings from the European Randomized Study of Screening for Prostate Cancer (ERSPC). Eur. Urol..

[33] Pinsky P.F., Prorok P.C., Yu K., Kramer B.S., Black A., Gohagan J.K., Crawford E.D., Grubb R.L., Andriole G.L. (2017). Extended mortality results for prostate cancer screening in the PLCO trial with median follow-up of 15 years. Cancer.

[34] Duffy M.J. (2020). Biomarkers for prostate cancer: Prostate-specific antigen and beyond. Clin. Chem. Lab. Med..

[35] Sanda M.G., Cadeddu J.A., Kirkby E., Chen R.C., Crispino T., Fontanarosa J., Freedland S.J., Greene K., Klotz L.H., Makarov D.V. (2018). Clinically Localized Prostate Cancer: AUA/ASTRO/SUO Guideline. Part I: Risk Stratification, Shared Decision Making, and Care Options. J. Urol..

[36] Smith R.A., Andrews K.S., Brooks D., Fedewa S.A., Manassaram-Baptiste D., Saslow D., Brawley O.W., Wender R.C. (2018). Cancer screening in the United States, 2018: A review of current American Cancer Society guidelines and current issues in cancer screening. CA Cancer J. Clin..

[37] Mottet N., Bellmunt J., Bolla M., Briers E., Cumberbatch M.G., De Santis M., Fossati N., Gross T., Henry A.M., Joniau S. (2017). EAU-ESTRO-SIOG Guidelines on Prostate Cancer. Part 1: Screening, Diagnosis, and Local Treatment with Curative Intent. Eur. Urol..

[38] Gandaglia G., Albers P., Abrahamsson P.A., Briganti A., Catto J.W., Chapple C.R., Montorsi F., Mottet N., Roobol M.J., Sønksen J. (2019). Structured Population-based Prostate-specific Antigen Screening for Prostate Cancer: The European Association of Urology Position in 2019. Eur. Urol..

[39] Vickers A.J., Eastham J.A., Scardino P.T., Lilja H. (2016). The Memorial Sloan Kettering Cancer Center Recommendations for Prostate Cancer Screening. Urology.

[40] Gasinska A., Jaszczynski J., Rychlik U., Łuczynska E., Pogodzinski M., Palaczynski M. (2020). Prognostic Significance of Serum PSA Level and Telomerase, VEGF and GLUT-1 Protein Expression for the Biochemical Recurrence in Prostate Cancer Patients after Radical Prostatectomy. Pathol. Oncol. Res..

[41] Liu D., Kuai Y., Zhu R., Zhou C., Tao Y., Han W., Chen Q. (2020). Prognosis of prostate cancer and bone metastasis pattern of patients: A SEER-based study and a local hospital based study from China. Sci. Rep..

[42] Vazquez Martinez M.A., Correa E., Jeurkar C., Shikdar S., Jain M.R., Topolsky D.L., Crilley P.A., Ward K.M., Styler M. (2018). The prognostic significance of PSA as an indicator of age standardized relative survival: An analysis of the SEER database 2004–2014. J. Clin. Oncol..

[43] Zheng Z., Zhou Z., Yan W., Zhou Y., Chen C., Li H., Ji Z. (2020). Tumor characteristics, treatments, and survival outcomes in prostate cancer patients with a PSA level <4 ng/mL: A population-based study. BMC Cancer.

[44] Ang M., Rajcic B., Foreman D., Moretti K., O’Callaghan M.E. (2016). Men presenting with prostate-specific antigen (PSA) values of over 100 ng/mL. BJU Int..

[45] Kang Y., Song P., Fang K., Yang B., Yang L., Zhou J., Wang L., Dong Q. (2020). Survival outcomes of low prostate-specific antigen levels and T stages in patients with high-grade prostate cancer: A population-matched study. J. Cancer.

[46] Mahal B.A., Yang D.D., Wang N.Q., Alshalalfa M., Davicioni E., Choeurng V., Schaeffer E.M., Ross A.E., Spratt D.E., Den R.B. (2018). Clinical and Genomic Characterization of Low-Prostate-specific Antigen, High-grade Prostate Cancer. Eur. Urol..

[47] McCormick B.Z., Mahmoud A.M., Williams S.B., Davis J.W. (2019). Biochemical recurrence after radical prostatectomy: Current status of its use as a treatment endpoint and early management strategies. Indian J. Urol..

[48] Zietman A.L., Christodouleas J.P., Shipley W.U. (2005). PSA bounces after neoadjuvant androgen deprivation and external beam radiation: Impact on definitions of failure. Int. J. Radiat. Oncol. Biol. Phys..

[49] Hanlon A.L., Pinover W.H., Horwitz E.M., Hanks G.E. (2001). Patterns and fate of PSA bouncing following 3D-CRT. Int. J. Radiat. Oncol. Biol. Phys..

[50] Abramowitz M.C., Li T., Buyyounouski M.K., Ross E., Uzzo R.G., Pollack A., Horwitz E.M. (2008). The Phoenix definition of biochemical failure predicts for overall survival in patients with prostate cancer. Cancer.

[51] Roach M., Hanks G., Thames H., Schellhammer P., Shipley W.U., Sokol G.H., Sandler H. (2006). Defining biochemical failure following radiotherapy with or without hormonal therapy in men with clinically localized prostate cancer: Recommendations of the RTOG-ASTRO Phoenix Consensus Conference. Int. J. Radiat. Oncol. Biol. Phys..

[52] Artibani W., Porcaro A.B., De Marco V., Cerruto M.A., Siracusano S. (2018). Management of biochemical recurrence after primary curative treatment for prostate cancer: A review. Urol. Int..

[53] Pound C.R., Partin A.W., Eisenberger M.A., Chan D.W., Pearson J.D., Walsh P.C. (1999). Natural history of progression after PSA elevation following radical prostatectomy. JAMA.

[54] Van den Broeck T., van den Bergh R.C., Arfi N., Gross T., Moris L., Briers E., Cumberbatch M., De Santis M., Tilki D., Fanti S. (2019). Prognostic Value of Biochemical Recurrence Following Treatment with Curative Intent for Prostate Cancer: A Systematic Review. Eur. Urol..

[55] Frydenberg M., Woo H.H. (2018). Early Androgen Deprivation Therapy Improves Survival, But How Do We Determine in Whom?. Eur. Urol..

[56] Brand D., Parker C. (2018). Management of Men with Prostate-specific Antigen Failure After Prostate Radiotherapy: The Case Against Early Androgen Deprivation. Eur. Urol..

[57] Garcia-Albeniz X., Chan J.M., Paciorek A., Logan R.W., Kenfield S.A., Cooperberg M.R., Carroll P.R., Hernán M.A. (2015). Immediate versus deferred initiation of androgen deprivation therapy in prostate cancer patients with PSA-only relapse. An observational follow-up study. Eur. J. Cancer.

[58] Duchesne G.M., Woo H.H., Bassett J.K., Bowe S.J., D’Este C., Frydenberg M., King M., Ledwich L., Loblaw A., Malone S. (2016). Timing of androgen-deprivation therapy in patients with prostate cancer with a rising PSA (TROG 03.06 and VCOG PR 01–03 [TOAD]): A randomised, multicentre, non-blinded, phase 3 trial. Lancet Oncol..

[59] Sasaki T., Sugimura Y. (2018). The Importance of Time to Prostate-Specific Antigen (PSA) Nadir after Primary Androgen Deprivation Therapy in Hormone-Naïve Prostate Cancer Patients. J. Clin. Med..

[60] Heidenreich A., Bastian P.J., Bellmunt J., Bolla M., Joniau S., van der Kwast T., Mason M., Matveev V., Wiegel T., Zattoni F. (2014). EAU Guidelines on Prostate Cancer. Part II: Treatment of Advanced, Relapsing, and Castration-Resistant Prostate Cancer. Eur. Urol..

[61] Morris M.J., Rumble R.B., Basch E., Hotte S.J., Loblaw A., Rathkopf D., Celano P., Bangs R., Milowsky M.I. (2018). Optimizing Anticancer Therapy in Metastatic Non-Castrate Prostate Cancer: American Society of Clinical Oncology Clinical Practice Guideline. J. Clin. Oncol..

[62] Hussain M., Tangen C.M., Higano C., Schelhammer P.F., Faulkner J., Crawford E.D., Wilding G., Akdas A., Small E.J., Donnelly B. (2006). Absolute prostate-specific antigen value after androgen deprivation is a strong independent predictor of survival in new metastatic prostate cancer: Data from Southwest Oncology Group Trial 9346 (INT-0162). J. Clin. Oncol..

[63] Harshman L.C., Chen Y.H., Liu G., Carducci M.A., Jarrard D., Dreicer R., Hahn N., Garcia J.A., Hussain M., Shevrin D. (2018). Seven-Month Prostate-Specific Antigen Is Prognostic in Metastatic Hormone-Sensitive Prostate Cancer Treated With Androgen Deprivation With or Without Docetaxel. J. Clin. Oncol. Off. J. Am. Soc. Clin. Oncol..

[64] Lorente D., Lozano R., de Velasco G., De Julian M., Rodrigo M., Sanchez A.L., Di Capua C., Castro E., Sanchez Hernandez A., Olmos D. (2019). Prognostic value of PSA progression in metastatic castration-resistant prostate cancer (mCRPC) patients (pts) treated in the COU-AA-302 trial. J. Clin. Oncol..

[65] España S., de Olza M.O., Sala N., Piulats J.M., Ferrandiz U., Etxaniz O., Heras L., Buisan O., Pardo J.C., Suarez J.F. (2020). PSA Kinetics as Prognostic Markers of Overall Survival in Patients with Metastatic Castration-Resistant Prostate Cancer Treated with Abiraterone Acetate. Cancer Manag. Res..

[66] Liu J.-M., Lin C.C., Liu K.L., Lin C.F., Chen B.Y., Chen T.H., Sun C.C., Wu C.T. (2020). Second-line Hormonal Therapy for the Management of Metastatic Castration-resistant Prostate Cancer: A Real-World Data Study Using a Claims Database. Sci. Rep..

[67] Scher H.I., Morris M.J., Stadler W.M., Higano C., Basch E., Fizazi K., Antonarakis E.S., Beer T.M., Carducci M.A., Chi K.N. (2016). Trial Design and Objectives for Castration-Resistant Prostate Cancer: Updated Recommendations From the Prostate Cancer Clinical Trials Working Group 3. J. Clin. Oncol..

[68] Ablin R.J., Soanes W.A., Bronson P., Witebsky E. (1970). Precipitating antigens of the normal human prostate. J. Reprod. Fertil..

[69] Lim K.B. (2017). Epidemiology of clinical benign prostatic hyperplasia. Asian J. Urol..

[70] Thompson I.M., Pauler D.K., Goodman P.J., Tangen C.M., Lucia M.S., Parnes H.L., Minasian L.M., Ford L.G., Lippman S.M., Crawford E.D. (2004). Prevalence of prostate cancer among men with a prostate-specific antigen level ≤ 4.0 ng per milliliter. N. Engl. J. Med..

[71] Thompson I.M., Ankerst D.P., Chi C., Lucia M.S., Goodman P.J., Crowley J.J., Parnes H.L., Coltman C.A. (2005). Operating characteristics of prostate-specific antigen in men with an initial PSA level of 3.0 ng/mL or lower. JAMA.

[72] Benson M.C., Whang I.S., Pantuck A., Ring K., Kaplan S.A., Olsson C.A., Cooner W.H. (1992). Prostate specific antigen density: A means of distinguishing benign prostatic hypertrophy and prostate cancer. J. Urol..

[73] Nordström T., Akre O., Aly M., Grönberg H., Eklund M. (2018). Prostate-specific antigen (PSA) density in the diagnostic algorithm of prostate cancer. Prostate Cancer Prostatic Dis..

[74] Yanai Y., Kosaka T., Hongo H., Matsumoto K., Shinojima T., Kikuchi E., Miyajima A., Mizuno R., Mikami S., Jinzaki M. (2018). Evaluation of prostate-specific antigen density in the diagnosis of prostate cancer combined with magnetic resonance imaging before biopsy in men aged 70 years and older with elevated PSA. Mol. Clin. Oncol..

[75] Aminsharifi A., Howard L., Wu Y., De Hoedt A., Bailey C., Freedland S.J., Polascik T.J. (2018). Prostate Specific Antigen Density as a Predictor of Clinically Significant Prostate Cancer When the Prostate Specific Antigen is in the Diagnostic Gray Zone: Defining the Optimum Cutoff Point Stratified by Race and Body Mass Index. J. Urol..

[76] Yusim I., Krenawi M., Mazor E., Novack V., Mabjeesh N.J. (2020). The use of prostate specific antigen density to predict clinically significant prostate cancer. Sci. Rep..

[77] Jue J.S., Barboza M.P., Prakash N.S., Venkatramani V., Sinha V.R., Pavan N., Nahar B., Kanabur P., Ahdoot M., Dong Y. (2017). Re-examining Prostate-specific Antigen (PSA) Density: Defining the Optimal PSA Range and Patients for Using PSA Density to Predict Prostate Cancer Using Extended Template Biopsy. Urology.

[78] Sfoungaristos S., Perimenis P. (2012). PSA density is superior than PSA and Gleason score for adverse pathologic features prediction in patients with clinically localized prostate cancer. Can. Urol. Assoc. J..

[79] Jin B.S., Kang S.H., Kim D.Y., Oh H.G., Kim C.I., Moon G.H., Kwon T.G., Park J.S. (2015). Pathological upgrading in prostate cancer patients eligible for active surveillance: Does prostate-specific antigen density matter?. Korean J. Urol..

[80] Ha Y.S., Yu J., Salmasi A.H., Patel N., Parihar J., Singer E.A., Kim J.H., Kwon T.G., Kim W.J., Kim I.Y. (2014). Prostate-specific antigen density toward a better cutoff to identify better candidates for active surveillance. Urology.

[81] Washington S.L., Baskin A.S., Ameli N., Nguyen H.G., Westphalen A.C., Shinohara K., Carroll P.R. (2020). MRI-Based Prostate-Specific Antigen Density Predicts Gleason Score Upgrade in an Active Surveillance Cohort. AJR Am. J. Roentgenol..

[82] Carter H.B., Pearson J.D., Metter E.J., Brant L.J., Chan D.W., Andres R., Fozard J.L., Walsh P.C. (1992). Longitudinal evaluation of prostate-specific antigen levels in men with and without prostate disease. JAMA.

[83] Vickers A.J., Brewster S.F. (2012). PSA Velocity and Doubling Time in Diagnosis and Prognosis of Prostate Cancer. Br. J. Med. Surg. Urol..

[84] Javaeed A., Ghauri S.K., Ibrahim A., Doheim M.F. (2020). Prostate-specific antigen velocity in diagnosis and prognosis of prostate cancer—A systematic review. Oncol. Rev..

[85] D’Amico A.V., Chen M.H., Roehl K.A., Catalona W.J. (2004). Preoperative PSA velocity and the risk of death from prostate cancer after radical prostatectomy. N. Engl. J. Med..

[86] Sengupta S., Myers R.P., Slezak J.M., Bergstralh E.J., Zincke H., Blute M.L. (2005). Preoperative prostate specific antigen doubling time and velocity are strong and independent predictors of outcomes following radical prostatectomy. J. Urol..

[87] Patel D.A., Presti J.C., McNeal J.E., Gill H., Brooks J.D., King C.R. (2005). Preoperative PSA velocity is an independent prognostic factor for relapse after radical prostatectomy. J. Clin. Oncol..

[88] Berger A.P., Deibl M., Strasak A., Bektic J., Pelzer A., Steiner H., Spranger R., Fritsche G., Bartsch G., Horninger W. (2006). Relapse after radical prostatectomy correlates with preoperative PSA velocity and tumor volume: Results from a screening population. Urology.

[89] D’Amico A.V., Renshaw A.A., Sussman B., Chen M.H. (2005). Pretreatment PSA velocity and risk of death from prostate cancer following external beam radiation therapy. JAMA.

[90] Palma D., Tyldesley S., Blood P., Liu M., Morris J., Pickles T., Prostate Cohort Outcomes Initiative (2007). Pretreatment PSA velocity as a predictor of disease outcome following radical radiation therapy. Int. J. Radiat. Oncol. Biol. Phys..

[91] Shimbo M., Tomioka S., Sasaki M., Shima T., Suzuki N., Murakami S., Nakatsu H., Shimazaki J. (2009). PSA Doubling Time as a Predictive Factor on Repeat Biopsy for Detection of Prostate Cancer. Jpn. J. Clin. Oncol..

[92] Takeuchi H., Ohori M., Tachibana M. (2017). Clinical significance of the prostate-specific antigen doubling time prior to and following radical prostatectomy to predict the outcome of prostate cancer. Mol. Clin. Oncol..

[93] Makarov D.V., Humphreys E.B., Mangold L.A., Carducci M.A., Partin A.W., Eisenberger M.A., Walsh P.C., Trock B.J. (2008). The natural history of men treated with deferred androgen deprivation therapy in whom metastatic prostate cancer developed following radical prostatectomy. J. Urol..

[94] Markowski M.C., Chen Y., Feng Z., Cullen J., Trock B.J., Suzman D., Antonarakis E.S., Paller C.J., Rosner I., Han M. (2019). PSA Doubling Time and Absolute PSA Predict Metastasis-free Survival in Men With Biochemically Recurrent Prostate Cancer After Radical Prostatectomy. Clin. Genitourin. Cancer.

[95] Jackson W.C., Johnson S.B., Li D., Foster C., Foster B., Song Y., Schipper M., Shilkrut M., Sandler H.M., Morgan T.M. (2013). A prostate-specific antigen doubling time of <6 months is prognostic for metastasis and prostate cancer-specific death for patients receiving salvage radiation therapy post radical prostatectomy. Radiat. Oncol..

[96] Whitney C.A., Howard L.E., Freedland S.J., DeHoedt A.M., Amling C.L., Aronson W.J., Cooperberg M.R., Kane C.J., Terris M.K., Daskivich T.J. (2017). Thresholds for PSA doubling time in men with non-metastatic castration-resistant prostate cancer. BJU Int..

[97] McLaren D.B., McKenzie M., Duncan G., Pickles T. (1998). Watchful waiting or watchful progression?. Cancer.

[98] Shariat S.F., Canto E.I., Kattan M.W., Slawin K.M. (2004). Beyond prostate-specific antigen: New serologic biomarkers for improved diagnosis and management of prostate cancer. Rev. Urol..

[99] Catalona W.J., Partin A.W., Slawin K.M., Brawer M.K., Flanigan R.C., Patel A., Richie J.P., DeKernion J.B., Walsh P.C., Scardino P.T. (1998). Use of the percentage of free prostate-specific antigen to enhance differentiation of prostate cancer from benign prostatic disease: A prospective multicenter clinical trial. JAMA.

[100] Partin A.W., Brawer M.K., Bartsch G., Horninger W., Taneja S.S., Lepor H., Babaian R., Childs S.J., Stamey T., Fritsche H.A. (2003). Complexed prostate specific antigen improves specificity for prostate cancer detection: Results of a prospective multicenter clinical trial. J. Urol..

[101] Prestigiacomo A.F., Lilja H., Pettersson K., Wolfert R.L., Stamey T.A. (1996). A comparison of the free fraction of serum prostate specific antigen in men with benign and cancerous prostates: The best case scenario. J. Urol..

[102] Piironen T., Pettersson K., Suonpää M., Stenman U.H., Oesterling J.E., Lövgren T., Lilja H. (1996). In vitro stability of free prostate-specific antigen (PSA) and prostate-specific antigen (PSA) complexed to α1-antichymotrypsin in blood samples. Urology.

[103] Ornstein D.K., Rao G.S., Smith D.S., Ratliff T.L., Basler J.W., Catalona W.J. (1997). Effect of digital rectal examination and needle biopsy on serum total and percentage of free prostate specific antigen levels. J. Urol..

[104] Stephan C., Lein M., Jung K., Schnorr D., Loening S.A. (1997). The influence of prostate volume on the ratio of free to total prostate specific antigen in serum of patients with prostate carcinoma and benign prostate hyperplasia. Cancer.

[105] Huang Y., Li Z.Z., Huang Y.L., Song H.J., Wang Y.J. (2018). Value of free/total prostate-specific antigen (f/t PSA) ratios for prostate cancer detection in patients with total serum prostate-specific antigen between 4 and 10 ng/mL: A meta-analysis. Medicine.

[106] Oto J., Fernández-Pardo Á., Royo M., Hervás D., Martos L., Vera-Donoso C.D., Martínez M., Heeb M.J., España F., Medina P. (2020). A predictive model for prostate cancer incorporating PSA molecular forms and age. Sci. Rep..

[107] Ayyıldız S.N., Ayyıldız A. (2014). PSA, PSA derivatives, proPSA and prostate health index in the diagnosis of prostate cancer. Turk. J. Urol..

[108] Ferro M., De Cobelli O., Lucarelli G., Porreca A., Busetto G.M., Cantiello F., Damiano R., Autorino R., Musi G., Vartolomei M.D. (2020). Beyond PSA: The Role of Prostate Health Index (phi). Int. J. Mol. Sci..

[109] Chan T.Y., Mikolajczyk S.D., Lecksell K., Shue M.J., Rittenhouse H.G., Partin A.W., Epstein J.I. (2003). Immunohistochemical staining of prostate cancer with monoclonal antibodies to the precursor of prostate-specific antigen. Urology.

[110] Jansen F.H., van Schaik R.H., Kurstjens J., Horninger W., Klocker H., Bektic J., Wildhagen M.F., Roobol M.J., Bangma C.H., Bartsch G. (2010). Prostate-specific antigen (PSA) isoform p2PSA in combination with total PSA and free PSA improves diagnostic accuracy in prostate cancer detection. Eur. Urol..

[111] Lazzeri M., Haese A., De La Taille A., Redorta J.P., McNicholas T., Lughezzani G., Scattoni V., Bini V., Freschi M., Sussman A. (2013). Serum isoform [-2]proPSA derivatives significantly improve prediction of prostate cancer at initial biopsy in a total PSA range of 2–10 ng/mL: A multicentric European study. Eur. Urol..

[112] Stephan C., Vincendeau S., Houlgatte A., Cammann H., Jung K., Semjonow A. (2013). Multicenter evaluation of [-2]proprostate-specific antigen and the prostate health index for detecting prostate cancer. Clin. Chem..

[113] Filella X., Giménez N. (2013). Evaluation of [-2]proPSA and Prostate Health Index (phi) for the detection of prostate cancer: A systematic review and meta-analysis. Clin. Chem. Lab. Med..

[114] Boegemann M., Stephan C., Cammann H., Vincendeau S., Houlgatte A., Jung K., Blanchet J.S., Semjonow A. (2016). The percentage of prostate-specific antigen (PSA) isoform [-2]proPSA and the Prostate Health Index improve the diagnostic accuracy for clinically relevant prostate cancer at initial and repeat biopsy compared with total PSA and percentage free PSA in men aged ≤65 years. BJU Int..

[115] Schulze A., Christoph F., Sachs M., Schroeder J., Stephan C., Schostak M., Koenig F. (2020). Use of the Prostate Health Index and Density in 3 Outpatient Centers to Avoid Unnecessary Prostate Biopsies. Urol. Int..

[116] Catalona W.J., Partin A.W., Sanda M.G., Wei J.T., Klee G.G., Bangma C.H., Slawin K.M., Marks L.S., Loeb S., Broyles D.L. (2011). A multicenter study of [-2]pro-prostate specific antigen combined with prostate specific antigen and free prostate specific antigen for prostate cancer detection in the 2.0 to 10.0 ng/mL prostate specific antigen range. J. Urol..

[117] Loeb S., Sanda M.G., Broyles D.L., Shin S.S., Bangma C.H., Wei J.T., Partin A.W., Klee G.G., Slawin K.M., Marks L.S. (2015). The prostate health index selectively identifies clinically significant prostate cancer. J. Urol..

[118] De La Calle C., Patil D., Wei J.T., Scherr D.S., Sokoll L., Chan D.W., Siddiqui J., Mosquera J.M., Rubin M.A., Sanda M.G. (2015). Multicenter evaluation of the prostate health index to detect aggressive prostate cancer in biopsy naive men. J. Urol..

[119] Novak V., Vesely S., Luksanová H., Prusa R., Capoun O., Fiala V., Dolejsová O., Sedlacková H., Kucera R., Stejskal J. (2020). Preoperative prostate health index predicts adverse pathology and Gleason score upgrading after radical prostatectomy for prostate cancer. BMC Urol..

[120] Tosoian J.J., Druskin S.C., Andreas D., Mullane P., Chappidi M., Joo S., Ghabili K., Agostino J., Macura K.J., Carter H.B. (2017). Use of the Prostate Health Index for detection of prostate cancer: Results from a large academic practice. Prostate Cancer Prostatic Dis..

[121] Olleik G., Kassouf W., Aprikian A., Hu J., Vanhuyse M., Cury F., Peacock S., Bonnevier E., Palenius E., Dragomir A. (2018). Evaluation of New Tests and Interventions for Prostate Cancer Management: A Systematic Review. J. Natl. Compr. Canc. Netw..

[122] Tosoian J.J., Druskin S.C., Andreas D., Mullane P., Chappidi M., Joo S., Ghabili K., Mamawala M., Agostino J., Carter H.B. (2017). Prostate Health Index density improves detection of clinically significant prostate cancer. BJU Int..

[123] Druskin S.C., Tosoian J.J., Young A., Collica S., Srivastava A., Ghabili K., Macura K.J., Carter H.B., Partin A.W., Sokoll L.J. (2018). Combining Prostate Health Index density, magnetic resonance imaging and prior negative biopsy status to improve the detection of clinically significant prostate cancer. BJU Int..

[124] Gnanapragasam V.J., Burling K., George A., Stearn S., Warren A., Barrett T., Koo B., Gallagher F.A., Doble A., Kastner C. (2016). The Prostate Health Index adds predictive value to multi-parametric MRI in detecting significant prostate cancers in a repeat biopsy population. Sci. Rep..

[125] White J., Shenoy B.V., Tutrone R.F., Karsh L.I., Saltzstein D.R., Harmon W.J., Broyles D.L., Roddy T.E., Lofaro L.R., Paoli C.J. (2018). Clinical utility of the Prostate Health Index (phi) for biopsy decision management in a large group urology practice setting. Prostate Cancer Prostatic Dis..

[126] Huang D., Wu Y., Lin X., Xu D., Na R., Xu J. (2020). Cost-Effectiveness Analysis of Prostate Health Index in Decision Making for Initial Prostate Biopsy. Front. Oncol..

[127] Teoh J.Y.-C., Leung C.H., Wang M.H., Chiu P.K.F., Yee C.H., Ng C.F., Wong M.C.S. (2020). The cost-effectiveness of prostate health index for prostate cancer detection in Chinese men. Prostate Cancer Prostatic Dis..

[128] Lughezzani G., Lazzeri M., Buffi N.M., Abrate A., Mistretta F.A., Hurle R., Pasini L., Castaldo L., De Zorzi S.Z., Peschechera R. (2015). Preoperative prostate health index is an independent predictor of early biochemical recurrence after radical prostatectomy: Results from a prospective single-center study. Urol. Oncol..

[129] Maxeiner A., Kilic E., Matalon J., Friedersdorff F., Miller K., Jung K., Stephan C., Busch J. (2017). The prostate health index PHI predicts oncological outcome and biochemical recurrence after radical prostatectomy—Analysis in 437 patients. Oncotarget.

[130] Chiu P.K., Ng C.F., Semjonow A., Zhu Y., Vincendeau S., Houlgatte A., Lazzeri M., Guazzoni G., Stephan C., Haese A. (2019). A Multicentre Evaluation of the Role of the Prostate Health Index (PHI) in Regions with Differing Prevalence of Prostate Cancer: Adjustment of PHI Reference Ranges is Needed for European and Asian Settings. Eur. Urol..

[131] Semjonow A., Köpke T., Eltze E., Pepping-Schefers B., Bürgel H., Darte C. (2010). Pre-analytical in-vitro stability of [-2]proPSA in blood and serum. Clin. Biochem..

[132] Hong S.K. (2014). Kallikreins as Biomarkers for Prostate Cancer. BioMed Res. Int..

[133] Vickers A.J., Cronin A.M., Aus G., Pihl C.G., Becker C., Pettersson K., Scardino P.T., Hugosson J., Lilja H. (2008). A panel of kallikrein markers can reduce unnecessary biopsy for prostate cancer: Data from the European Randomized Study of Prostate Cancer Screening in Göteborg, Sweden. BMC Med..

[134] Gupta A., Roobol M.J., Savage C.J., Peltola M., Pettersson K., Scardino P.T., Vickers A.J., Schröder F.H., Lilja H. (2010). A four-kallikrein panel for the prediction of repeat prostate biopsy: Data from the European Randomized Study of Prostate Cancer screening in Rotterdam, Netherlands. Br. J. Cancer.

[135] Vickers A., Cronin A., Roobol M., Savage C., Peltola M., Pettersson K., Scardino P.T., Schröder F., Lilja H. (2010). Reducing unnecessary biopsy during prostate cancer screening using a four-kallikrein panel: An independent replication. J. Clin. Oncol..

[136] Vickers A.J., Cronin A.M., Roobol M.J., Savage C.J., Peltola M., Pettersson K., Scardino P.T., Schröder F.H., Lilja H. (2010). A four-kallikrein panel predicts prostate cancer in men with recent screening: Data from the European Randomized Study of Screening for Prostate Cancer, Rotterdam. Clin. Cancer Res..

[137] Parekh D.J., Punnen S., Sjoberg D.D., Asroff S.W., Bailen J.L., Cochran J.S., Concepcion R., David R.D., Deck K.B., Dumbadze I. (2015). A multi-institutional prospective trial in the USA confirms that the 4Kscore accurately identifies men with high-grade prostate cancer. Eur. Urol..

[138] Nordström T., Vickers A., Assel M., Lilja H., Grönberg H., Eklund M. (2015). Comparison Between the Four-kallikrein Panel and Prostate Health Index for Predicting Prostate Cancer. Eur. Urol..

[139] Verbeek J.F.M., Bangma C.H., Kweldam C.F., van der Kwast T.H., Kümmerlin I.P., van Leenders G.J., Roobol M.J. (2019). Reducing unnecessary biopsies while detecting clinically significant prostate cancer including cribriform growth with the ERSPC Rotterdam risk calculator and 4Kscore. Urol. Oncol..

[140] Braun K., Sjoberg D.D., Vickers A.J., Lilja H., Bjartell A.S. (2016). A four-kallikrein panel predicts high-grade cancer on biopsy: Independent validation in a community cohort. Eur. Urol..

[141] Assel M., Sjöblom L., Murtola T.J., Talala K., Kujala P., Stenman U.H., Taari K., Auvinen A., Vickers A., Visakorpi T. (2019). A Four-kallikrein Panel and β-Microseminoprotein in Predicting High-grade Prostate Cancer on Biopsy: An Independent Replication from the Finnish Section of the European Randomized Study of Screening for Prostate Cancer. Eur. Urol. Focus.

[142] Lin D.W., Newcomb L.F., Brown M.D., Sjoberg D.D., Dong Y., Brooks J.D., Carroll P.R., Cooperberg M., Dash A., Ellis W.J. (2017). Evaluating the four kallikrein panel of the 4kscore for prediction of high-grade prostate cancer in men in the Canary Prostate Active Surveillance Study. Eur. Urol..

[143] Zappala S.M., Dong Y., Linder V., Reeve M., Sjoberg D.D., Mathur V., Roberts R., Okrongly D., Newmark J., Sant G. (2017). The 4Kscore blood test accurately identifies men with aggressive prostate cancer prior to prostate biopsy with or without DRE information. Int. J. Clin. Pract..

[144] Punnen S., Nahar B., Soodana-Prakash N., Koru-Sengul T., Stoyanova R., Pollack A., Kava B., Gonzalgo M.L., Ritch C.R., Parekh D.J. (2018). Optimizing patient’s selection for prostate biopsy: A single institution experience with multi-parametric MRI and the 4Kscore test for the detection of aggressive prostate cancer. PLoS ONE.

[145] Darst B.F., Chou A., Wan P., Pooler L., Sheng X., Vertosick E.A., Conti D.V., Wilkens L.R., Le Marchand L., Vickers A.J. (2020). The Four-Kallikrein Panel Is Effective in Identifying Aggressive Prostate Cancer in a Multiethnic Population. Cancer Epidemiol. Biomark. Prev..

[146] Konety B., Zappala S.M., Parekh D.J., Osterhout D., Schock J., Chudler R.M., Oldford G.M., Kernen K.M., Hafron J. (2015). The 4Kscore^®^ Test Reduces Prostate Biopsy Rates in Community and Academic Urology Practices. Rev. Urol..

[147] Voigt J.D., Dong Y., Linder V., Zappala S. (2017). Use of the 4Kscore test to predict the risk of aggressive prostate cancer prior to prostate biopsy: Overall cost savings and improved quality of care to the us healthcare system. Rev. Urol..

[148] Voigt J.D., Zappala S.M., Vaughan E.D., Wein A.J. (2014). The Kallikrein Panel for prostate cancer screening: Its economic impact. Prostate.

[149] Stattin P., Vickers A.J., Sjoberg D.D., Johansson R., Granfors T., Johansson M., Pettersson K., Scardino P.T., Hallmans G., Lilja H. (2015). Improving the specificity of screening for lethal prostate cancer using prostate-specific antigen and a panel of kallikrein markers: A nested case–control study. Eur. Urol..

[150] Al Olama A.A., Kote-Jarai Z., Berndt S.I., Conti D.V., Schumacher F., Han Y., Benlloch S., Hazelett D.J., Wang Z., Saunders E. (2014). A meta-analysis of 87,040 individuals identifies 23 new susceptibility loci for prostate cancer. Nat. Genet..

[151] Aly M., Wiklund F., Xu J., Isaacs W.B., Eklund M., D’Amato M., Adolfsson J., Grönberg H. (2011). Polygenic risk score improves prostate cancer risk prediction: Results from the Stockholm-1 cohort study. Eur. Urol..

[152] Kader A.K., Sun J., Reck B.H., Newcombe P.J., Kim S.T., Hsu F.C., D’Agostino R.B., Tao S., Zhang Z., Turner A.R. (2012). Potential impact of adding genetic markers to clinical parameters in predicting prostate biopsy outcomes in men following an initial negative biopsy: Findings from the REDUCE trial. Eur. Urol..

[153] Grönberg H., Adolfsson J., Aly M., Nordström T., Wiklund P., Brandberg Y., Thompson J., Wiklund F., Lindberg J., Clements M. (2015). Prostate cancer screening in men aged 50–69 years (STHLM3): A prospective population-based diagnostic study. Lancet Oncol..

[154] Eklund M., Nordström T., Aly M., Adolfsson J., Wiklund P., Brandberg Y., Thompson J., Wiklund F., Lindberg J., Presti J.C. (2018). The Stockholm-3 (STHLM3) Model can Improve Prostate Cancer Diagnostics in Men Aged 50–69 yr Compared with Current Prostate Cancer Testing. Eur. Urol. Focus.

[155] Ström P., Nordström T., Aly M., Egevad L., Grönberg H., Eklund M. (2018). The Stockholm-3 Model for Prostate Cancer Detection: Algorithm Update, Biomarker Contribution, and Reflex Test Potential. Eur. Urol..

[156] Möller A., Olsson H., Grönberg H., Eklund M., Aly M., Nordström T. (2019). The Stockholm3 blood-test predicts clinically-significant cancer on biopsy: Independent validation in a multi-center community cohort. Prostate Cancer Prostatic Dis..

[157] Viste E., Vinje C.A., Lid T.G., Skeie S., Evjen-Olsen Ø., Nordström T., Thorsen O., Gilje B., Janssen E.A., Kjosavik S.R. (2020). Effects of replacing PSA with Stockholm3 for diagnosis of clinically significant prostate cancer in a healthcare system—The Stavanger experience. Scand. J. Prim. Health Care.

[158] Varki A. (2017). Biological roles of glycans. Glycobiology.

[159] Munkley J., Elliott D.J. (2016). Hallmarks of glycosylation in cancer. Oncotarget.

[160] Srinivasan S., Stephens C., Wilson E., Panchadsaram J., DeVoss K., Koistinen H., Stenman U.H., Brook M.N., Buckle A.M., Klein R.J. (2019). Prostate Cancer Risk-Associated Single-Nucleotide Polymorphism Affects Prostate-Specific Antigen Glycosylation and Its Function. Clin. Chem..

[161] Drake R.R., Jones E.E., Powers T.W., Nyalwidhe J.O. (2015). Altered glycosylation in prostate cancer. Adv. Cancer Res..

[162] Pinho S.S., Reis C.A. (2015). Glycosylation in cancer: Mechanisms and clinical implications. Nat. Rev. Cancer..

[163] Peracaula R., Tabarés G., Royle L., Harvey D.J., Dwek R.A., Rudd P.M., de Llorens R. (2003). Altered glycosylation pattern allows the distinction between prostate-specific antigen (PSA) from normal and tumor origins. Glycobiology.

[164] Meany D.L., Zhang Z., Sokoll L.J., Zhang H., Chan D.W. (2009). Glycoproteomics for prostate cancer detection: Changes in serum PSA glycosylation patterns. J. Proteome Res..

[165] Ishikawa T., Yoneyama T., Tobisawa Y., Hatakeyama S., Kurosawa T., Nakamura K., Narita S., Mitsuzuka K., Duivenvoorden W., Pinthus J.H. (2017). An Automated Micro-Total Immunoassay System for Measuring Cancer-Associated α2,3-linked Sialyl N-Glycan-Carrying Prostate-Specific Antigen May Improve the Accuracy of Prostate Cancer Diagnosis. Int. J. Mol. Sci..

[166] Llop E., Ferrer-Batallé M., Barrabés S., Guerrero P.E., Ramírez M., Saldova R., Rudd P.M., Aleixandre R.N., Comet J., de Llorens R. (2016). Improvement of prostate cancer diagnosis by detecting PSA glycosylation-specific changes. Theranostics.

[167] Ferrer-Batallé M., Llop E., Ramírez M., Aleixandre R.N., Saez M., Comet J., De Llorens R., Peracaula R. (2017). Comparative Study of Blood-Based Biomarkers, α2,3-Sialic Acid PSA and PHI, for High-Risk Prostate Cancer Detection. Int. J. Mol. Sci..

[168] Massie C.E., Mills I.G., Lynch A.G. (2017). The importance of DNA methylation in prostate cancer development. J. Steroid Biochem. Mol. Biol..

[169] Kirby M.K., Ramaker R.C., Roberts B.S., Lasseigne B.N., Gunther D.S., Burwell T.C., Davis N.S., Gulzar Z.G., Absher D.M., Cooper S.J. (2017). Genome-wide DNA methylation measurements in prostate tissues uncovers novel prostate cancer diagnostic biomarkers and transcription factor binding patterns. BMC Cancer.

[170] O’Reilly E., Tuzova A.V., Walsh A.L., Russell N.M., O’Brien O., Kelly S., Dhomhnallain O.N., DeBarra L., Dale C.M., Brugman R. (2019). epiCaPture: A Urine DNA Methylation Test for Early Detection of Aggressive Prostate Cancer. JCO Precis. Oncol..

[171] Leyten G.H., Hessels D., Smit F.P., Jannink S.A., de Jong H., Melchers W.J., Cornel E.B., de Reijke T.M., Vergunst H., Kil P. (2015). Identification of a Candidate Gene Panel for the Early Diagnosis of Prostate Cancer. Clin. Cancer Res..

[172] Van Neste L., Hendriks R.J., Dijkstra S., Trooskens G., Cornel E.B., Jannink S.A., de Jong H., Hessels D., Smit F.P., Melchers W.J. (2016). Detection of High-grade Prostate Cancer Using a Urinary Molecular Biomarker-Based Risk Score. Eur. Urol..

[173] Haese A., Trooskens G., Steyaert S., Hessels D., Brawer M., Vlaeminck-Guillem V., Ruffion A., Tilki D., Schalken J., Groskopf J. (2019). Multicenter Optimization and Validation of a 2-Gene mRNA Urine Test for Detection of Clinically Significant Prostate Cancer before Initial Prostate Biopsy. J. Urol..

[174] Hendriks R.J., van der Leest M.M., Dijkstra S., Barentsz J.O., Van Criekinge W., Hulsbergen-van de Kaa C.A., Schalken J.A., Mulders P.F., van Oort I.M. (2017). A urinary biomarker-based risk score correlates with multiparametric MRI for prostate cancer detection. Prostate.

[175] Busetto G.M., Del Giudice F., Maggi M., De Marco F., Porreca A., Sperduti I., Magliocca F.M., Salciccia S., Chung B.I., De Berardinis E. (2020). Prospective assessment of two-gene urinary test with multiparametric magnetic resonance imaging of the prostate for men undergoing primary prostate biopsy. World J. Urol..

[176] Govers T.M., Hessels D., Vlaeminck-Guillem V., Schmitz-Dräger B.J., Stief C.G., Martinez-Ballesteros C., Ferro M., Borque-Fernando A., Rubio-Briones J., Sedelaar J.M. (2019). Cost-effectiveness of SelectMDx for prostate cancer in four European countries: A comparative modeling study. Prostate Cancer Prostatic Dis..

[177] Dijkstra S., Govers T.M., Hendriks R.J., Schalken J.A., Van Criekinge W., Van Neste L., Grutters J.P., Sedelaar J.P.M., van Oort I.M. (2017). Cost-effectiveness of a new urinary biomarker-based risk score compared to standard of care in prostate cancer diagnostics—A decision analytical model. BJU Int..

[178] Sathianathen N.J., Kuntz K.M., Alarid-Escudero F., Lawrentschuk N.L., Bolton D.M., Murphy D.G., Weight C.J., Konety B.R. (2018). Incorporating Biomarkers into the Primary Prostate Biopsy Setting: A Cost-Effectiveness Analysis. J. Urol..

[179] Hoyer G., Crawford E.D., Arangua P., Stanton W., La Rosa F.G., Poage W., Lucia M.S., van Bokhoven A., Werahera P.N. (2019). SelectMDx versus Prostate Health Index in the identification of high-grade prostate cancer. J. Clin. Oncol..

[180] Fasulo V., de la Calle C.M., Cowan J.E., Herlemann A., Chu C., Gadzinski A.J., Au Yeung R., Saita A., Cooperberg M.R., Shinohara K. (2020). Clinical utility of biomarkers 4K score, SelectMDx and ExoDx with MRI for the detection of high-grade prostate cancer. J. Clin. Oncol..

[181] Pepe P., Dibenedetto G., Pepe L., Pennisi M. (2020). Multiparametric MRI Versus SelectMDx Accuracy in the Diagnosis of Clinically Significant PCa in Men Enrolled in Active Surveillance. In Vivo.

[182] Zheng J. (2013). Oncogenic chromosomal translocations and human cancer (review). Oncol. Rep..

[183] Tomlins S.A., Rhodes D.R., Perner S., Dhanasekaran S.M., Mehra R., Sun X.W., Varambally S., Cao X., Tchinda J., Kuefer R. (2005). Recurrent fusion of TMPRSS2 and ETS transcription factor genes in prostate cancer. Science.

[184] Prensner J.R., Chinnaiyan A.M. (2009). Oncogenic gene fusions in epithelial carcinomas. Curr. Opin. Genet. Dev..

[185] Han B., Mehra R., Lonigro R.J., Wang L., Suleman K., Menon A., Palanisamy N., Tomlins S.A., Chinnaiyan A.M., Shah R.B. (2009). Fluorescence in situ hybridization study shows association of PTEN deletion with ERG rearrangement during prostate cancer progression. Mod. Pathol..

[186] Magi-Galluzzi C., Tsusuki T., Elson P., Simmerman K., LaFargue C., Esgueva R., Klein E., Rubin M.A., Zhou M. (2011). TMPRSS2-ERG gene fusion prevalence and class are significantly different in prostate cancer of Caucasian, African-American and Japanese patients. Prostate.

[187] Mosquera J.M., Perner S., Genega E.M., Sanda M., Hofer M.D., Mertz K.D., Paris P.L., Simko J., Bismar T.A., Ayala G. (2008). Characterization of TMPRSS2-ERG fusion high-grade prostatic intraepithelial neoplasia and potential clinical implications. Clin. Cancer Res..

[188] Hessels D., Smit F.P., Verhaegh G.W., Witjes J.A., Cornel E.B., Schalken J.A. (2007). Detection of TMPRSS2-ERG fusion transcripts and prostate cancer antigen 3 in urinary sediments may improve diagnosis of prostate cancer. Clin. Cancer Res..

[189] Tomlins S.A., Aubin S.M., Siddiqui J., Lonigro R.J., Sefton-Miller L., Miick S., Williamsen S., Hodge P., Meinke J., Blase A. (2011). Urine TMPRSS2:ERG fusion transcript stratifies prostate cancer risk in men with elevated serum PSA. Sci. Transl. Med..

[190] Demichelis F., Fall K., Perner S., Andrén O., Schmidt F., Setlur S.R., Hoshida Y., Mosquera J.M., Pawitan Y., Lee C. (2007). TMPRSS2:ERG gene fusion associated with lethal prostate cancer in a watchful waiting cohort. Oncogene.

[191] Lapointe J., Kim Y.H., Miller M.A., Li C., Kaygusuz G., van de Rijn M., Huntsman D.G., Brooks J.D., Pollack J.R. (2007). A variant TMPRSS2 isoform and ERG fusion product in prostate cancer with implications for molecular diagnosis. Mod. Pathol..

[192] Winnes M., Lissbrant E., Damber J.E., Stenman G. (2007). Molecular genetic analyses of the TMPRSS2-ERG and TMPRSS2-ETV1 gene fusions in 50 cases of prostate cancer. Oncol. Rep..

[193] Rice K.R., Chen Y., Ali A., Whitman E.J., Blase A., Ibrahim M., Elsamanoudi S., Brassell S., Furusato B., Stingle N. (2010). Evaluation of the ETS-related gene mRNA in urine for the detection of prostate cancer. Clin. Cancer Res..

[194] Fine S.W., Gopalan A., Leversha M.A., Al-Ahmadie H.A., Tickoo S.K., Zhou Q., Satagopan J.M., Scardino P.T., Gerald W.L., Reuter V.E. (2010). TMPRSS2-ERG gene fusion is associated with low Gleason scores and not with high-grade morphological features. Mod. Pathol..

[195] Gopalan A., Leversha M.A., Satagopan J.M., Zhou Q., Al-Ahmadie H.A., Fine S.W., Eastham J.A., Scardino P.T., Scher H.I., Tickoo S.K. (2009). TMPRSS2-ERG gene fusion is not associated with outcome in patients treated by prostatectomy. Cancer Res..

[196] Pettersson A., Graff R.E., Bauer S.R., Pitt M.J., Lis R.T., Stack E.C., Martin N.E., Kunz L., Penney K.L., Ligon A.H. (2012). The TMPRSS2:ERG rearrangement, ERG expression, and prostate cancer outcomes: A cohort study and meta-analysis. Cancer Epidemiol. Biomark. Prev..

[197] Vader P., Breakefield X.O., Wood M.J. (2014). Extracellular vesicles: Emerging targets for cancer therapy. Trends Mol. Med..

[198] Kahlert C., Kalluri R. (2013). Exosomes in tumor microenvironment influence cancer progression and metastasis. J. Mol. Med..

[199] Tavoosidana G., Ronquist G., Darmanis S., Yan J., Carlsson L., Wu D., Conze T., Ek P., Semjonow A., Eltze E. (2011). Multiple recognition assay reveals prostasomes as promising plasma biomarkers for prostate cancer. Proc. Natl. Acad. Sci. USA.

[200] Nilsson J., Skog J., Nordstrand A., Baranov V., Mincheva-Nilsson L., Breakefield X.O., Widmark A. (2009). Prostate cancer-derived urine exosomes: A novel approach to biomarkers for prostate cancer. Br. J. Cancer.

[201] Wang Wei-Lin W., Sorokin I., Aleksic I., Fisher H., Kaufman R.P., Winer A., McNeill B., Gupta R., Tilki D., Fleshner N. (2020). Expression of Small Noncoding RNAs in Urinary Exosomes Classifies Prostate Cancer into Indolent and Aggressive Disease. J. Urol..

[202] Stewart G.D., Van Neste L., Delvenne P., Delrée P., Delga A., McNeill S.A., O’Donnell M., Clark J., Van Criekinge W., Bigley J. (2013). Clinical utility of an epigenetic assay to detect occult prostate cancer in histopathologically negative biopsies: Results of the MATLOC study. J. Urol..

[203] Yang B., Bhusari S., Kueck J., Weeratunga P., Wagner J., Leverson G., Huang W., Jarrard D.F. (2013). Methylation profiling defines an extensive field defect in histologically normal prostate tissues associated with prostate cancer. Neoplasia.

[204] Partin A.W., Van Neste L., Klein E.A., Marks L.S., Gee J.R., Troyer D.A., Rieger-Christ K., Jones J.S., Magi-Galluzzi C., Mangold L.A. (2014). Clinical Validation of an Epigenetic Assay to Predict Negative Histopathological Results in Repeat Prostate Biopsies. J. Urol..

[205] Van Neste L., Partin A.W., Stewart G.D., Epstein J.I., Harrison D.J., Van Criekinge W. (2016). Risk score predicts high-grade prostate cancer in DNA-methylation positive, histopathologically negative biopsies. Prostate.

[206] Waterhouse R.L., Van Neste L., Moses K.A., Barnswell C., Silberstein J.L., Jalkut M., Tutrone R., Sylora J., Anglade R., Murdock M. (2019). Evaluation of an Epigenetic Assay for Predicting Repeat Prostate Biopsy Outcome in African American Men. Urology.

[207] Bussemakers M.J., Van Bokhoven A., Verhaegh G.W., Smit F.P., Karthaus H.F., Schalken J.A., Debruyne F.M., Ru N., Isaacs W.B. (1999). Dd3:: A new prostate-specific gene, highly overexpressed in prostate cancer. Cancer Res..

[208] De Kok J.B., Verhaegh G.W., Roelofs R.W., Hessels D., Kiemeney L.A., Aalders T.W., Swinkels D.W., Schalken J.A. (2002). DD3PCA3, a very sensitive and specific marker to detect prostate tumors. Cancer Res..

[209] Hessels D., Gunnewiek J.M.K., van Oort I., Karthaus H.F., van Leenders G.J., van Balken B., Kiemeney L.A., Witjes J.A. (2003). and Schalken, J.A. DD3(PCA3)-based molecular urine analysis for the diagnosis of prostate cancer. Eur. Urol..

[210] Tinzl M., Marberger M., Horvath S., Chypre C. (2004). DD3PCA3 RNA analysis in urine—A new perspective for detecting prostate cancer. Eur. Urol..

[211] Groskopf J., Aubin S.M., Deras I.L., Blase A., Bodrug S., Clark C., Brentano S., Mathis J., Pham J., Meyer T. (2006). APTIMA PCA3 molecular urine test: Development of a method to aid in the diagnosis of prostate cancer. Clin. Chem..

[212] Auprich M., Bjartell A., Chun F.K.H., de la Taille A., Freedland S.J., Haese A., Schalken J., Stenzl A., Tombal B., van der Poel H. (2011). Contemporary role of prostate cancer antigen 3 in the management of prostate cancer. Eur. Urol..

[213] van Poppel H., Haese A., Graefen M., de la Taille A., Irani J., de Reijke T., Remzi M., Marberger M. (2012). The relationship between Prostate CAncer gene 3 (PCA3) and prostate cancer significance. BJU Int..

[214] Rodríguez S.V.M., García-Perdomo H.A. (2020). Diagnostic accuracy of prostate cancer antigen 3 (PCA3) prior to first prostate biopsy: A systematic review and meta-analysis. Can. Urol. Assoc. J..

[215] Wei J.T., Feng Z., Partin A.W., Brown E., Thompson I., Sokoll L., Chan D.W., Lotan Y., Kibel A.S., Busby J.E. (2014). Can urinary PCA3 supplement PSA in the early detection of prostate cancer?. J. Clin. Oncol..

[216] Gittelman M.C., Hertzman B., Bailen J., Williams T., Koziol I., Henderson R.J., Efros M., Bidair M., Ward J.F. (2013). PCA3 molecular urine test as a predictor of repeat prostate biopsy outcome in men with previous negative biopsies: A prospective multicenter clinical study. J. Urol..

[217] Pepe P., Aragona F. (2011). PCA3 Score vs PSA Free/Total Accuracy in Prostate Cancer Diagnosis at Repeat Saturation Biopsy. Anticancer Res..

[218] Leyten G.H., Wierenga E.A., Sedelaar J.P., Van Oort I.M., Futterer J.J., Barentsz J.O., Schalken J.A., Mulders P.F. (2013). Value of PCA3 to predict biopsy outcome and its potential role in selecting patients for multiparametric MRI. Int. J. Mol. Sci..

[219] Kaufmann S., Bedke J., Gatidis S., Hennenlotter J., Kramer U., Notohamiprodjo M., Nikolaou K., Stenzl A., Kruck S. (2016). Prostate cancer gene 3 (PCA3) is of additional predictive value in patients with PI-RADS grade III (intermediate) lesions in the MR-guided re-biopsy setting for prostate cancer. World J. Urol..

[220] De Luca S., Passera R., Cattaneo G., Manfredi M., Mele F., Fiori C., Bollito E., Cirillo S., Porpiglia F. (2016). High prostate cancer gene 3 (PCA3) scores are associated with elevated Prostate Imaging Reporting and Data System (PI-RADS) grade and biopsy Gleason score, at magnetic resonance imaging/ultrasonography fusion software-based targeted prostate biopsy after a previous negative standard biopsy. BJU Int..

[221] Yamkamon V., Htoo K.P.P., Yainoy S., Suksrichavalit T., Tangchaikeeree T., Eiamphungporn W. (2020). Urinary PCA3 detection in prostate cancer by magnetic nanoparticles coupled with colorimetric enzyme-linked oligonucleotide assay. Excli. J..

[222] Soares J.C., Soares A.C., Rodrigues V.C., Melendez M.E., Santos A.C., Faria E.F., Reis R.M., Carvalho A.L., Oliveira O.N. (2019). Detection of the Prostate Cancer Biomarker PCA3 with Electrochemical and Impedance-Based Biosensors. ACS Appl. Mater. Interfaces.

[223] Fu X., Wen J., Li J., Lin H., Liu Y., Zhuang X., Tian C., Chen L. (2019). Highly sensitive detection of prostate cancer specific PCA3 mimic DNA using SERS-based competitive lateral flow assay. Nanoscale.

[224] Htoo K.P.P., Yamkamon V., Yainoy S., Suksrichavalit T., Viseshsindh W., Eiamphungporn W. (2019). Colorimetric detection of PCA3 in urine for prostate cancer diagnosis using thiol-labeled PCR primer and unmodified gold nanoparticles. Clin. Chim. Acta.

[225] Merola R., Tomao L., Antenucci A., Sperduti I., Sentinelli S., Masi S., Mandoj C., Orlandi G., Papalia R., Guaglianone S. (2015). PCA3 in prostate cancer and tumor aggressiveness detection on 407 high-risk patients: A National Cancer Institute experience. J. Exp. Clin. Cancer Res..

[226] Chevli K.K., Duff M., Walter P., Yu C., Capuder B., Elshafei A., Malczewski S., Kattan M.W., Jones J.S. (2014). Urinary PCA3 as a predictor of prostate cancer in a cohort of 3073 men undergoing initial prostate biopsy. J. Urol..

[227] Haese A., de la Taille A., Van Poppel H., Marberger M., Stenzl A., Mulders P.F., Huland H., Abbou C.C., Remzi M., Tinzl M. (2008). Clinical utility of the PCA3 urine assay in European men scheduled for repeat biopsy. Eur. Urol..

[228] Tosoian J.J., Patel H.D., Mamawala M., Landis P., Wolf S., Elliott D.J., Epstein J.I., Carter H.B., Ross A.E., Sokoll L.J. (2017). Longitudinal assessment of urinary PCA3 for predicting prostate cancer grade reclassification in favorable-risk men during active surveillance. Prostate Cancer Prostatic Dis..

[229] Chunhua L., Zhao H., Zhao H., Lu Y., Wu J., Gao Z., Li G., Zhang Y., Wang K. (2018). Clinical Significance of Peripheral Blood PCA3 Gene Expression in Early Diagnosis of Prostate Cancer. Transl. Oncol..

[230] Hessels D., van Gils M.P., van Hooij O., Jannink S.A., Witjes J.A., Verhaegh G.W., Schalken J.A. (2010). Predictive value of PCA3 in urinary sediments in determining clinico-pathological characteristics of prostate cancer. Prostate.

[231] Foj L., Milà M., Mengual L., Luque P., Alcaraz A., Jiménez W., Filella X. (2014). Real-time PCR PCA3 assay is a useful test measured in urine to improve prostate cancer detection. Clin. Chim. Acta.

[232] Scattoni V., Lazzeri M., Lughezzani G., De Luca S., Passera R., Bollito E., Randone D., Abdollah F., Capitanio U., Larcher A. (2013). Head-to-head comparison of prostate health index and urinary PCA3 for predicting cancer at initial or repeat biopsy. J. Urol..

[233] Cantiello F., Russo G.I., Ferro M., Cicione A., Cimino S., Favilla V., Perdonà S., Bottero D., Terracciano D., De Cobelli O. (2015). Prognostic accuracy of Prostate Health Index and urinary Prostate Cancer Antigen 3 in predicting pathologic features after radical prostatectomy. Urol. Oncol..

[234] Salami S.S., Schmidt F., Laxman B., Regan M.M., Rickman D.S., Scherr D., Bueti G., Siddiqui J., Tomlins S.A., Wei J.T. (2013). Combining urinary detection of TMPRSS2: ERG and PCA3 with serum PSA to predict diagnosis of prostate cancer. Urologic Oncology: Seminars and Original Investigations.

[235] Tomlins S.A., Day J.R., Lonigro R.J., Hovelson D.H., Siddiqui J., Kunju L.P., Dunn R.L., Meyer S., Hodge P., Groskopf J. (2016). Urine TMPRSS2:ERG Plus PCA3 for Individualized Prostate Cancer Risk Assessment. Eur. Urol..

[236] McKiernan J., Donovan M.J., O’Neill V., Bentink S., Noerholm M., Belzer S., Skog J., Kattan M.W., Partin A., Andriole G. (2016). A novel urine exosome gene expression assay to predict high-grade prostate cancer at initial biopsy. JAMA Oncol..

[237] McKiernan J., Donovan M.J., Margolis E., Partin A., Carter B., Brown G., Torkler P., Noerholm M., Skog J., Shore N. (2018). A Prospective Adaptive Utility Trial to Validate Performance of a Novel Urine Exosome Gene Expression Assay to Predict High-grade Prostate Cancer in Patients with Prostate-specific Antigen 2–10 ng/mL at Initial Biopsy. Eur. Urol..

[238] McKiernan J., Noerholm M., Tadigotla V., Kumar S., Torkler P., Sant G., Alter J., Donovan M.J., Skog J. (2020). A urine-based Exosomal gene expression test stratifies risk of high-grade prostate Cancer in men with prior negative prostate biopsy undergoing repeat biopsy. BMC Urol..

[239] Tutrone R., Donovan M.J., Torkler P., Tadigotla V., McLain T., Noerholm M., Skog J., McKiernan J. (2020). Clinical utility of the exosome based ExoDx Prostate(IntelliScore) EPI test in men presenting for initial Biopsy with a PSA 2–10 ng/mL. Prostate Cancer Prostatic Dis..

[240] Dakubo G.D., Jakupciak J.P., Birch-Machin M.A., Parr R.L. (2007). Clinical implications and utility of field cancerization. Cancer Cell Int..

[241] Maki J., Robinson K., Reguly B., Alexander J., Wittock R., Aguirre A., Diamandis E.P., Escott N., Skehan A., Prowse O. (2008). Mitochondrial genome deletion aids in the identification of false- and true-negative prostate needle core biopsy specimens. Am. J. Clin. Pathol..

[242] Robinson K., Creed J., Reguly B., Powell C., Wittock R., Klein D., Maggrah A., Klotz L., Parr R.L., Dakubo G.D. (2010). Accurate prediction of repeat prostate biopsy outcomes by a mitochondrial DNA deletion assay. Prostate Cancer Prostatic Dis..

[243] Legisi L., DeSa E., Qureshi M.N. (2016). Use of the Prostate Core Mitomic Test in Repeated Biopsy Decision-Making: Real-World Assessment of Clinical Utility in a Multicenter Patient Population. Am. Health Drug Benefits.

[244] Eschrich S.A., Fulp W.J., Pawitan Y., Foekens J.A., Smid M., Martens J.W., Echevarria M., Kamath V., Lee J.H., Harris E.E. (2012). Validation of a Radiosensitivity Molecular Signature in Breast Cancer. Clin. Cancer Res..

[245] Weichselbaum R.R., Ishwaran H., Yoon T., Nuyten D.S., Baker S.W., Khodarev N., Su A.W., Shaikh A.Y., Roach P., Kreike B. (2008). An interferon-related gene signature for DNA damage resistance is a predictive marker for chemotherapy and radiation for breast cancer. Proc. Natl. Acad. Sci. USA.

[246] Pitroda S.P., Pashtan I.M., Logan H.L., Budke B., Darga T.E., Weichselbaum R.R., Connell P.P. (2014). DNA repair pathway gene expression score correlates with repair proficiency and tumor sensitivity to chemotherapy. Sci. Transl. Med..

[247] Paik S., Tang G., Shak S., Kim C., Baker J., Kim W., Cronin M., Baehner F.L., Watson D., Bryant J. (2006). Gene expression and benefit of chemotherapy in women with node-negative, estrogen receptor-positive breast cancer. J. Clin. Oncol..

[248] Klein E.A., Cooperberg M.R., Magi-Galluzzi C., Simko J.P., Falzarano S.M., Maddala T., Chan J.M., Li J., Cowan J.E., Tsiatis A.C. (2014). A 17-gene assay to predict prostate cancer aggressiveness in the context of Gleason grade heterogeneity, tumor multifocality, and biopsy undersampling. Eur. Urol..

[249] Eggener S., Karsh L.I., Richardson T., Shindel A.W., Lu R., Rosenberg S., Goldfischer E., Korman H., Bennett J., Newmark J. (2019). A 17-gene Panel for Prediction of Adverse Prostate Cancer Pathologic Features: Prospective Clinical Validation and Utility. Urology.

[250] Cullen J., Rosner I.L., Brand T.C., Zhang N., Tsiatis A.C., Moncur J., Ali A., Chen Y., Knezevic D., Maddala T. (2015). A biopsy-based 17-gene genomic prostate score predicts recurrence after radical prostatectomy and adverse surgical pathology in a racially diverse population of men with clinically low-and intermediate-risk prostate cancer. Eur. Urol..

[251] CMoschovas M.C., Chew C., Bhat S., Sandri M., Rogers T., Dell’Oglio P., Roof S., Reddy S., Sighinolfi M.C., Rocco B. (2021). Association Between Oncotype DX Genomic Prostate Score and Adverse Tumor Pathology After Radical Prostatectomy. Eur. Urol. Focus.

[252] Kornberg Z., Cooperberg M.R., Cowan J.E., Chan J., Shinohara K., Simko J.P., Tenggara I., Carroll P.R. (2019). A 17-Gene Genomic Prostate Score as a Predictor of Adverse Pathology in Men on Active Surveillance. J. Urol..

[253] Eeden S.K.V.D., Lu R., Zhang N., Quesenberry C.P., Shan J., Han J.S., Tsiatis A.C., Leimpeter A.D., Lawrence H.J., Febbo P.G. (2018). A Biopsy-based 17-gene Genomic Prostate Score as a Predictor of Metastases and Prostate Cancer Death in Surgically Treated Men with Clinically Localized Disease. Eur. Urol..

[254] Cullen J., Kuo H.-C., Shan J., Lu R., Aboushwareb T., Eeden S.K.V.D. (2020). The 17-Gene Genomic Prostate Score Test as a Predictor of Outcomes in Men with Unfavorable Intermediate Risk Prostate Cancer. Urology.

[255] Chang E.M., Punglia R.S., Steinberg M.L., Raldow A.C. (2019). Cost Effectiveness of the Oncotype DX Genomic Prostate Score for Guiding Treatment Decisions in Patients With Early Stage Prostate Cancer. Urology.

[256] Albala D., Kemeter M.J., Febbo P.G., Lu R., John V., Stoy D., Denes B., McCall M., Shindel A.W., Dubeck F. (2016). Health Economic Impact and Prospective Clinical Utility of Oncotype DX^®^ Genomic Prostate Score. Rev. Urol..

[257] Lin D.W., Zheng Y., McKenney J.K., Brown M.D., Lu R., Crager M., Boyer H., Tretiakova M., Brooks J.D., Dash A. (2020). 17-Gene Genomic Prostate Score Test Results in the Canary Prostate Active Surveillance Study (PASS) Cohort. J. Clin. Oncol..

[258] Greenland Nancy Y., Zhang L., Cowan J.E., Carroll P.R., Stohr B.A., Simko J.P. (2019). Correlation of a Commercial Genomic Risk Classifier with Histological Patterns in Prostate Cancer. J. Urol..

[259] Cuzick J., Swanson G., Fisher G., Brothman A.R., Berney D., E Reid J., Mesher D., Speights V., Stankiewicz E., Foster C.S. (2011). Prognostic value of an RNA expression signature derived from cell cycle proliferation genes in patients with prostate cancer: A retrospective study. Lancet Oncol..

[260] (2018). NICE, NICE Advice—Prolaris gene expression assay for assessing long-term risk of prostate cancer progression: © NICE (2016) Prolaris gene expression assay for assessing long-term risk of prostate cancer progression. BJU Int..

[261] Cuzick J., Stone S.B., Fisher G., Yang Z.H., North B.V.,  Berney D., Beltran L.E., Greenberg D.S., Moller H., On behalf of the Transatlantic Prostate Group (2015). Validation of an RNA cell cycle progression score for predicting death from prostate cancer in a conservatively managed needle biopsy cohort. Br. J. Cancer.

[262] Lin D.W., Crawford E.D., Keane T., Evans B., Reid J., Rajamani S., Brown K., Gutin A., Tward J., Scardino P. (2018). Identification of men with low-risk biopsy-confirmed prostate cancer as candidates for active surveillance. Urol. Oncol..

[263] Bishoff J.T., Freedland S.J., Gerber L., Tennstedt P., Reid J., Welbourn W., Graefen M., Sangale Z., Tikishvili E., Park J. (2014). Prognostic utility of the cell cycle progression score generated from biopsy in men treated with prostatectomy. J. Urol..

[264] Cooperberg M.R., Simko J.P., Cowan J.E., Reid J.E., Djalilvand A., Bhatnagar S., Gutin A., Lanchbury J.S., Swanson G., Stone S. (2013). Validation of a cell-cycle progression gene panel to improve risk stratification in a contemporary prostatectomy cohort. J. Clin. Oncol..

[265] Tosoian J.J., Chappidi M.R., Bishoff J.T., Freedland S.J., Reid J., Brawer M., Stone S., Schlomm T., Ross A.E. (2017). Prognostic utility of biopsy-derived cell cycle progression score in patients with National Comprehensive Cancer Network low-risk prostate cancer undergoing radical prostatectomy: Implications for treatment guidance. BJU Int..

[266] Léon P., Cancel-Tassin G., Drouin S., Audouin M., Varinot J., Comperat E., Cathelineau X., Rozet F., Vaessens C., Stone S. (2018). Comparison of cell cycle progression score with two immunohistochemical markers (PTEN and Ki-67) for predicting outcome in prostate cancer after radical prostatectomy. World J. Urol..

[267] Freedland S.J., Gerber L., Reid J., Welbourn W., Tikishvili E., Park J., Younus A., Gutin A., Sangale Z., Lanchbury J.S. (2013). Prognostic utility of cell cycle progression score in men with prostate cancer after primary external beam radiation therapy. Int. J. Radiat. Oncol. Biol. Phys..

[268] Koch M.O., Cho J.S., Kaimakliotis H.Z., Cheng L., Sangale Z., Brawer M., Welbourn W., Reid J., Stone S. (2016). Use of the cell cycle progression (CCP) score for predicting systemic disease and response to radiation of biochemical recurrence. Cancer Biomark.

[269] Canter D.J., Freedland S., Rajamani S., Latsis M., Variano M., Halat S., Tward J., Cohen T., Stone S., Schlomm T. (2020). Analysis of the prognostic utility of the cell cycle progression (CCP) score generated from needle biopsy in men treated with definitive therapy. Prostate Cancer Prostatic Dis..

[270] Shore N.D., Kella N., Moran B., Boczko J., Bianco F.J., Crawford E.D., Davis T., Roundy K.M., Rushton K., Grier C. (2016). Impact of the Cell Cycle Progression Test on Physician and Patient Treatment Selection for Localized Prostate Cancer. J. Urol..

[271] Kaul S., Wojno K.J., Stone S., Evans B., Bernhisel R., Meek S., D’Anna R.E., Ferguson J., Glaser J., Morgan T.M. (2019). Clinical outcomes in men with prostate cancer who selected active surveillance using a clinical cell cycle risk score. Per. Med..

[272] Crawford E.D., Scholz M.C., Kar A.J., Fegan J.E., Haregewoin A., Kaldate R.R., Brawer M.K. (2014). Cell cycle progression score and treatment decisions in prostate cancer: Results from an ongoing registry. Curr. Med. Res. Opin..

[273] Gustavsen G., Taylor K., Cole D., Gullet L., Lewine N. (2020). Health economic impact of a biopsy-based cell cycle gene expression assay in localized prostate cancer. Future Oncol..

[274] Health Quality Ontario (2017). Prolaris Cell Cycle Progression Test for Localized Prostate Cancer: A Health Technology Assessment. Ont. Health Technol. Assess. Ser..

[275] Shipitsin M., E Small C., Choudhury S., Giladi E., Friedlander S.F., Nardone J., Hussain S., Hurley A.D., Ernst C., E Huang Y. (2014). Identification of proteomic biomarkers predicting prostate cancer aggressiveness and lethality despite biopsy-sampling error. Br. J. Cancer.

[276] Blume-Jensen P., Berman D., Rimm D.L., Shipitsin M., Putzi M., Nifong T.P., Small C., Choudhury S., Capela T., Coupal L. (2015). Development and clinical validation of an in situ biopsy-based multimarker assay for risk stratification in prostate cancer. Clin. Cancer Res..

[277] Erho N., Crisan A., Vergara I.A., Mitra A.P., Ghadessi M., Buerki C., Bergstralh E.J., Kollmeyer T., Fink S., Haddad Z. (2013). Discovery and validation of a prostate cancer genomic classifier that predicts early metastasis following radical prostatectomy. PLoS ONE.

[278] Klein E.A., Yousefi K., Haddad Z., Choeurng V., Buerki C., Stephenson A.J., Li J., Kattan M., Magi-Galluzzi C., Davicioni E. (2015). A genomic classifier improves prediction of metastatic disease within 5 years after surgery in node-negative high-risk prostate cancer patients managed by radical prostatectomy without adjuvant therapy. Eur. Urol..

[279] Broeck T.V.D., Moris L., Gevaert T., Tosco L., Smeets E., Fishbane N., Liu Y., Helsen C., Margrave J., Buerki C. (2019). Validation of the Decipher Test for Predicting Distant Metastatic Recurrence in Men with High-risk Nonmetastatic Prostate Cancer 10 Years After Surgery. Eur. Urol. Oncol..

[280] Karnes R.J., Bergstralh E.J., Davicioni E., Ghadessi M., Buerki C., Mitra A.P., Crisan A., Erho N., Vergara I., Lam L.L. (2013). Validation of a genomic classifier that predicts metastasis following radical prostatectomy in an at risk patient population. J. Urol..

[281] Spratt D.E., Yousefi K., Deheshi S., Ross A.E., Den R., Schaeffer E.M., Trock B.J., Zhang J., Glass A.G., Dicker A.P. (2017). Individual Patient-Level Meta-Analysis of the Performance of the Decipher Genomic Classifier in High-Risk Men After Prostatectomy to Predict Development of Metastatic Disease. J. Clin. Oncol..

[282] Alshalalfa M., Crisan A., Vergara I., Ghadessi M., Buerki C., Erho N., Yousefi K., Sierocinski T., Haddad Z., Black P.C. (2015). Clinical and genomic analysis of metastatic prostate cancer progression with a background of postoperative biochemical recurrence. BJU Int..

[283] Spratt D.E., Zhang J., Santiago-Jiménez M., Dess R.T., Davis J.W., Den R.B., Dicker A.P., Kane C.J., Pollack A., Stoyanova R. (2018). Development and Validation of a Novel Integrated Clinical-Genomic Risk Group Classification for Localized Prostate Cancer. J. Clin. Oncol..

[284] Klein E.A., Haddad Z., Yousefi K., Lam L.L., Wang Q., Choeurng V., Palmer-Aronsten B., Buerki C., Davicioni E., Li J. (2016). Decipher genomic classifier measured on prostate biopsy predicts metastasis risk. Urology.

[285] Nguyen P.L., Haddad Z., Ross A.E., Martin N.E., Deheshi S., Lam L.L., Chelliserry J., Tosoian J.J., Lotan T.L., Spratt D.E. (2017). Ability of a Genomic Classifier to Predict Metastasis and Prostate Cancer-specific Mortality after Radiation or Surgery based on Needle Biopsy Specimens. Eur. Urol..

[286] Jambor I., Falagario U., Ratnani P., Msc I.M.P., Demir K., Merisaari H., Sobotka S., Haines G.K., Martini A., Beksac A.T. (2020). Prediction of biochemical recurrence in prostate cancer patients who underwent prostatectomy using routine clinical prostate multiparametric MRI and decipher genomic score. J. Magn. Reson. Imaging.

[287] Ross A.E., Feng F.Y., Ghadessi M., Erho N., Crisan A., Buerki C., Sundi D., Mitra A.P., Vergara I., Thompson D.J.S. (2014). A genomic classifier predicting metastatic disease progression in men with biochemical recurrence after prostatectomy. Prostate Cancer Prostatic Dis..

[288] Kim H.L., Li P., Huang H.-C., Deheshi S., Marti T., Knudsen B., Abou-Ouf H., Alam R., Lotan T., Lam L.L.C. (2019). Validation of the Decipher Test for predicting adverse pathology in candidates for prostate cancer active surveillance. Prostate Cancer Prostatic Dis..

[289] Den R.B., Yousefi K., Trabulsi E.J., Abdollah F., Choeurng V., Feng F.Y., Dicker A.P., Lallas C.D., Gomella L.G., Davicioni E. (2015). Genomic classifier identifies men with adverse pathology after radical prostatectomy who benefit from adjuvant radiation therapy. J. Clin. Oncol..

[290] Michalopoulos S.N., Michalopoulos S.N., Kella N., Payne R., Yohannes P., Singh A., Hettinger C., Yousefi K., Hornberger J., On behalf of the PRO-ACT Study Group (2014). Influence of a genomic classifier on post-operative treatment decisions in high-risk prostate cancer patients: Results from the PRO-ACT study. Curr. Med. Res. Opin..

[291] Badani K.K., Thompson D.J., Brown G., Holmes D., Kella N., Albala D., Singh A., Buerki C., Davicioni E., Hornberger J. (2015). Effect of a genomic classifier test on clinical practice decisions for patients with high-risk prostate cancer after surgery. BJU Int..

[292] Badani K., Thompson D.J.S., Buerki C., Davicioni E., Garrison J., Ghadessi M., Mitra A.P., Wood P.J., Hornberger J. (2013). Impact of a genomic classifier of metastatic risk on postoperative treatment recommendations for prostate cancer patients: A report from the DECIDE study group. Oncotarget.

[293] Jairath N.K., Pra A.D., Vince R., Dess R.T., Jackson W.C., Tosoian J.J., McBride S.M., Zhao S.G., Berlin A., Mahal B.A. (2021). A Systematic Review of the Evidence for the Decipher Genomic Classifier in Prostate Cancer. Eur. Urol..

[294] Yerushalmi R., Woods R., Ravdin P.M., Hayes M.M., Gelmon K.A. (2010). Ki67 in breast cancer: Prognostic and predictive potential. Lancet Oncol..

[295] Li R., Heydon K., Hammond M.E., Grignon D.J., Roach M., Wolkov H.B., Sandler H.M., Shipley W.U., Pollack A. (2004). Ki-67 staining index predicts distant metastasis and survival in locally advanced prostate cancer treated with radiotherapy: An analysis of patients in radiation therapy oncology group protocol 86–10. Clin. Cancer Res..

[296] Pollack A., DeSilvio M., Khor L.-Y., Li R., Al-Saleem T., Hammond M., Venkatesan V., Lawton C., Roach M., Shipley W. (2004). Ki-67 staining is a strong predictor of distant metastasis and mortality for men with prostate cancer treated with radiotherapy plus androgen deprivation: Radiation Therapy Oncology Group Trial 92–02. J. Clin. Oncol..

[297] Verhoven B., Yan Y., Ritter M., Khor L.-Y., Hammond E., Jones C., Amin M., Bahary J.-P., Zeitzer K., Pollack A. (2013). Ki-67 is an independent predictor of metastasis and cause-specific mortality for prostate cancer patients treated on Radiation Therapy Oncology Group (RTOG) 94–08. Int. J. Radiat. Oncol. Biol. Phys..

[298] Mathieu R., Shariat S.F., Seitz C., Karakiewicz P.I., Fajkovic H., Sun M., Lotan Y., Scherr D., Tewari A., Montorsi F. (2015). Multi-institutional validation of the prognostic value of Ki-67 labeling index in patients treated with radical prostatectomy. World J. Urol..

[299] Berlin A., Berlin A., Castro-Mesta J.F., Rodriguez-Romo L., Hernandez-Barajas D., González-Guerrero J.F., Rodríguez-Fernández I.A., González-Conchas G., Verdines-Perez A., Vera-Badillo F.E. (2017). Prognostic role of Ki-67 score in localized prostate cancer: A systematic review and meta-analysis. Urol. Oncol..

[300] Epstein J.I., Amin M.B., Fine S.W., Algaba F., Aron M., Baydar D.E., Beltran A.L., Brimo F., Cheville J.C., Colecchia M. (2021). The 2019 Genitourinary Pathology Society (GUPS) White Paper on Contemporary Grading of Prostate Cancer. Arch. Pathol. Lab. Med..

[301] Mesko S., Kupelian P., Demanes D.J., Huang J., Wang P.C., Kamrava M. (2013). Quantifying the ki-67 heterogeneity profile in prostate cancer. Prostate Cancer.

[302] Van der Kwast T.H. (2014). Prognostic prostate tissue biomarkers of potential clinical use. Virchows Arch..

[303] Esteller M. (2011). Non-coding RNAs in human disease. Nat. Rev. Genet..

[304] Liu C., Kelnar K., Liu B., Chen X., Calhoun-Davis T., Li H., Patrawala L., Yan H., Jeter C., Honorio S. (2011). The microRNA miR-34a inhibits prostate cancer stem cells and metastasis by directly repressing CD44. Nat. Med..

[305] Kong D., Heath E., Chen W., Cher M.L., Powell I., Heilbrun L., Li Y., Ali S., Sethi S., Hassan O. (2012). Loss of let-7 up-regulates EZH2 in prostate cancer consistent with the acquisition of cancer stem cell signatures that are attenuated by BR-DIM. PLoS ONE.

[306] Mitchell P.S., Parkin R.K., Kroh E.M., Fritz B.R., Wyman S.K., Pogosova-Agadjanyan E.L., Peterson A., Noteboom J., O’Briant K.C., Allen A. (2008). Circulating microRNAs as stable blood-based markers for cancer detection. Proc. Natl. Acad. Sci. USA.

[307] Mihelich B.L., Maranville J.C., Nolley R., Peehl D.M., Nonn L. (2015). Elevated serum microRNA levels associate with absence of high-grade prostate cancer in a retrospective cohort. PLoS ONE.

[308] Al-Qatati A., Akrong C., Stevic I., Pantel K., Awe J., Saranchuk J., Drachenberg D., Mai S., Schwarzenbach H. (2017). Plasma micro RNA signature is associated with risk stratification in prostate cancer patients. Int. J. Cancer.

[309] Salido-Guadarrama A.I., Morales-Montor J.G., Rangel-Escareño C., Langley E., Peralta-Zaragoza O., Cruz Colin J.L., Rodriguez-Dorantes M. (2016). Urinary microRNA-based signature improves accuracy of detection of clinically relevant prostate cancer within the prostate-specific antigen grey zone. Mol. Med. Rep..

[310] Foj L., Ferrer F., Serra M., Arévalo A., Gavagnach M., Gimenez N., Filella X. (2017). Exosomal and non-exosomal urinary miRNAs in prostate cancer detection and prognosis. Prostate.

[311] Wise H.M., Hermida M.A., Leslie N.R. (2017). Prostate cancer, PI3K, PTEN and prognosis. Clin. Sci..

[312] Taylor B.S., Schultz N., Hieronymus H., Gopalan A., Xiao Y., Carver B.S., Arora V.K., Kaushik P., Cerami E., Reva B. (2010). Integrative genomic profiling of human prostate cancer. Cancer Cell.

[313] Lotan T.L., Wei W., Ludkovski O., Morais C.L., Guedes L.B., Jamaspishvili T., Lopez K., Hawley S.T., Feng Z., Fazli L. (2016). Analytic validation of a clinical-grade PTEN immunohistochemistry assay in prostate cancer by comparison with PTEN FISH. Mod. Pathol..

[314] Lotan T.L., Carvalho F.L., Peskoe S.B., Hicks J.L., Good J., Fedor H.L., Humphreys E.B., Han M., Platz E.A., Squire J. (2015). PTEN loss is associated with upgrading of prostate cancer from biopsy to radical prostatectomy. Mod. Pathol..

[315] Yoshimoto M., Cunha I.W., A Coudry R., Fonseca F.P., Torres C.H., Soares F.A., A Squire J. (2007). FISH analysis of 107 prostate cancers shows that PTEN genomic deletion is associated with poor clinical outcome. Br. J. Cancer.

[316] Köksal I.T., Dirice E., Yasar D., Sanlioglu A.D., Ciftcioglu A., Gulkesen K.H., O Ozes N., Baykara M., Luleci G., Sanlioglu S. (2004). The assessment of PTEN tumor suppressor gene in combination with Gleason scoring and serum PSA to evaluate progression of prostate carcinoma. Urologic Oncology: Seminars and Original Investigations.

[317] Lotan T., Gurel B., Sutcliffe S., Esopi D., Liu W., Xu J., Hicks J.L., Park B.H., Humphreys E., Partin A.W. (2011). PTEN protein loss by immunostaining: Analytic validation and prognostic indicator for a high risk surgical cohort of prostate cancer patients. Clin. Cancer Res..

[318] Chaux A., Peskoe S.B., Gonzalez-Roibon N., Schultz L., Albadine R., Hicks J., De Marzo A.M., A Platz E., Netto G.J. (2012). Loss of PTEN expression is associated with increased risk of recurrence after prostatectomy for clinically localized prostate cancer. Mod. Pathol..

[319] Xie H., Xie B., Liu C., Wang J., Xu Y. (2017). Association of PTEN expression with biochemical recurrence in prostate cancer: Results based on previous reports. OncoTargets Ther..

[320] Ferraldeschi R., Rodrigues D.N., Riisnaes R., Miranda S., Figueiredo I., Rescigno P., Ravi P., Pezaro C., Omlin A., Lorente D. (2015). PTEN protein loss and clinical outcome from castration-resistant prostate cancer treated with abiraterone acetate. Eur. Urol..

[321] Mulholland D.J., Tran L.M., Li Y., Cai H., Morim A., Wang S., Plaisier S., Garraway I.P., Huang J., Graeber T. (2011). Cell autonomous role of PTEN in regulating castration-resistant prostate cancer growth. Cancer Cell.

[322] Parker C.C., Clarke N.W., Cook A.D., Kynaston H.G., Petersen P.M., Catton C., Cross W., Logue J., Parulekar W., Payne H. (2020). Timing of radiotherapy after radical prostatectomy (RADICALS-RT): A randomised, controlled phase 3 trial. Lancet.

[323] Zhao S.G., Chang S.L., E Spratt D., Erho N., Yu M., Ashab H.A.-D., Alshalalfa M., Speers C., A Tomlins S., Davicioni E. (2016). Development and validation of a 24-gene predictor of response to postoperative radiotherapy in prostate cancer: A matched, retrospective analysis. Lancet Oncol..

[324] Zhang W., van Gent D.C., Incrocci L., van Weerden W.M., Nonnekens J. (2020). Role of the DNA damage response in prostate cancer formation, progression and treatment. Prostate Cancer Prostatic Dis..

[325] Cheng H.H. (2018). The resounding effect of DNA repair deficiency in prostate cancer. Urol. Oncol..

[326] Nizialek E., Antonarakis E.S. (2020). PARP Inhibitors in Metastatic Prostate Cancer: Evidence to Date. Cancer Manag. Res..

[327] Teyssonneau D., Margot H., Cabart M., Anonnay M., Sargos P., Vuong N.-S., Soubeyran I., Sevenet N., Roubaud G. (2021). Prostate cancer and PARP inhibitors: Progress and challenges. J. Hematol. Oncol..

[328] Fujita K., Nonomura N. (2019). Role of Androgen Receptor in Prostate Cancer: A Review. World J. Mens Health.

[329] Crawford E.D., Heidenreich A., Lawrentschuk N., Tombal B., Pompeo A.C.L., Mendoza-Valdes A., Miller K., Debruyne F.M.J., Klotz L. (2019). Androgen-targeted therapy in men with prostate cancer: Evolving practice and future considerations. Prostate Cancer Prostatic Dis..

[330] Coutinho I., Day T.K., Tilley W., Selth L.A. (2016). Androgen receptor signaling in castration-resistant prostate cancer: A lesson in persistence. Endocr. Relat. Cancer.

[331] Karantanos T., Evans C.P., Tombal B., Thompson T.C., Montironi R., Isaacs W.B. (2015). Understanding the mechanisms of androgen deprivation resistance in prostate cancer at the molecular level. Eur. Urol..

[332] Luo J. (2016). Development of AR-V7 as a putative treatment selection marker for metastatic castration-resistant prostate cancer. Asian J. Androl..

[333] Antonarakis E.S., Lu C., Wang H., Luber B., Nakazawa M., Roeser J.C., Chen Y., Mohammad T.A., Chen Y., Fedor H.L. (2014). AR-V7 and resistance to enzalutamide and abiraterone in prostate cancer. N. Engl. J. Med..

[334] Antonarakis E.S., Lu C., Luber B., Wang H., Chen Y., Zhu Y., Silberstein J.L., Taylor M.N., Maughan B.L., Denmeade S.R. (2017). Clinical Significance of Androgen Receptor Splice Variant-7 mRNA Detection in Circulating Tumor Cells of Men With Metastatic Castration-Resistant Prostate Cancer Treated With First- and Second-Line Abiraterone and Enzalutamide. J. Clin. Oncol..

[335] Armstrong A.J., Halabi S., Luo J., Nanus D.M., Giannakakou P., Szmulewitz R.Z., Danila D.C., Healy P., Anand M., Rothwell C.J. (2019). Prospective Multicenter Validation of Androgen Receptor Splice Variant 7 and Hormone Therapy Resistance in High-Risk Castration-Resistant Prostate Cancer: The PROPHECY Study. J. Clin. Oncol..

[336] Armstrong A.J., Luo J., Anand M., Antonarakis E.S., Nanus D.M., Giannakakou P., Szmulewitz R.Z., Danila D.C., Healy P., Berry W.R. (2020). AR-V7 and prediction of benefit with taxane therapy: Final analysis of PROPHECY. J. Clin. Oncol..

[337] Scher H.I., Lu D., Schreiber N.A., Louw J., Graf R.P., Vargas H.A., Johnson A., Jendrisak A., Bambury R., Danila D. (2016). Association of AR-V7 on Circulating Tumor Cells as a Treatment-Specific Biomarker With Outcomes and Survival in Castration-Resistant Prostate Cancer. JAMA Oncol..

[338] Sharp A., Welti J.C., Lambros M.B., Dolling D., Rodrigues D.N., Pope L., Aversa C., Figueiredo I., Fraser J., Ahmad Z. (2019). Clinical Utility of Circulating Tumour Cell Androgen Receptor Splice Variant-7 Status in Metastatic Castration-resistant Prostate Cancer. Eur. Urol..

[339] Surdacki G., Szudy-Szczyrek A., Gorący A., Chyl-Surdacka K., Hus M. (2019). The role of immune checkpoint inhibitors in prostate cancer. Ann. Agric. Environ. Med..

[340] Sharma P., Pachynski R.K., Narayan V., Fléchon A., Gravis G., Galsky M.D., Mahammedi H., Patnaik A., Subudhi S.K., Ciprotti M. (2020). Nivolumab Plus Ipilimumab for Metastatic Castration-Resistant Prostate Cancer: Preliminary Analysis of Patients in the CheckMate 650 Trial. Cancer Cell.

[341] Fizazi K., Drake C.G., Beer T.M., Kwon E.D., Scher H.I., Gerritsen W.R., Bossi A., Eertwegh A.J.V.D., Krainer M., Houede N. (2020). Final Analysis of the Ipilimumab Versus Placebo Following Radiotherapy Phase III Trial in Postdocetaxel Metastatic Castration-resistant Prostate Cancer Identifies an Excess of Long-term Survivors. Eur. Urol..

[342] Patel S.P., Kurzrock R. (2015). PD-L1 Expression as a Predictive Biomarker in Cancer Immunotherapy. Mol. Cancer Ther..

[343] Zhang T., Agarwal A., Almquist R.G., Runyambo D., Park S., Bronson E., Boominathan R., Rao C., Anand M., Oyekunle T. (2021). Expression of immune checkpoints on circulating tumor cells in men with metastatic prostate cancer. Biomark. Res..

[344] Wang L., Pan S., Zhu B., Yu Z., Wang W. (2021). Comprehensive analysis of tumour mutational burden and its clinical significance in prostate cancer. BMC Urol..

[345] Ryan M.J., Bose R. (2019). Genomic Alteration Burden in Advanced Prostate Cancer and Therapeutic Implications. Front. Oncol..

[346] Goodman A.M., Kato S., Bazhenova L., Patel S.P., Frampton G.M., Miller V., Stephens P.J., Daniels G.A., Kurzrock R. (2017). Tumor Mutational Burden as an Independent Predictor of Response to Immunotherapy in Diverse Cancers. Mol. Cancer Ther..

[347] Hellmann M.D., Nathanson T., Rizvi H., Creelan B.C., Sanchez-Vega F., Ahuja A., Ni A., Novik J.B., Mangarin L.M., Abu-Akeel M. (2018). Genomic Features of Response to Combination Immunotherapy in Patients with Advanced Non-Small-Cell Lung Cancer. Cancer Cell.

[348] Blazek D., Kohoutek J., Bartholomeeusen K., Johansen E., Hulinkova P., Luo Z., Cimermancic P., Ule J., Peterlin B.M. (2011). The Cyclin K/Cdk12 complex maintains genomic stability via regulation of expression of DNA damage response genes. Genes Dev..

[349] Wu Y.-M., Cieślik M., Lonigro R.J., Vats P., Reimers M.A., Cao X., Ning Y., Wang L., Kunju L.P., de Sarkar N. (2018). Inactivation of CDK12 Delineates a Distinct Immunogenic Class of Advanced Prostate Cancer. Cell.

[350] Antonarakis E.S., Velho P.I., Fu W., Wang H., Agarwal N., Santos V.S., Maughan B.L., Pili R., Adra N., Sternberg C.N. (2020). CDK12-Altered Prostate Cancer: Clinical Features and Therapeutic Outcomes to Standard Systemic Therapies, Poly (ADP-Ribose) Polymerase Inhibitors, and PD-1 Inhibitors. JCO Precis. Oncol..

[351] Salami S.S., Hovelson D.H., Kaplan J.B., Mathieu R., Udager A.M., Curci N.E., Lee M., Plouffe K.R., De La Vega L.L., Susani M. (2018). Transcriptomic heterogeneity in multifocal prostate cancer. JCI Insight.

[352] Wei L., Wang J., Lampert E., Schlanger S., DePriest A.D., Hu Q., Gomez E.C., Murakam M., Glenn S.T., Conroy J. (2017). Intratumoral and Intertumoral Genomic Heterogeneity of Multifocal Localized Prostate Cancer Impacts Molecular Classifications and Genomic Prognosticators. Eur. Urol..

[353] Alam S., Tortora J., Staff I., McLaughlin T., Wagner J. (2019). Prostate cancer genomics: Comparing results from three molecular assays. Can. J. Urol..

[354] Lehto T.K., Stürenberg C., Malén A., Erickson A.M., Koistinen H., Mills I.G., Rannikko A., Mirtti T. (2021). Transcript analysis of commercial prostate cancer risk stratification panels in hard-to-predict grade group 2–4 prostate cancers. Prostate.

[355] Murphy A.B., Carbunaru S., Nettey O.S., Gornbein C., Dixon M.A., Macias V., Sharifi R., Kittles R.A., Yang X., Kajdacsy-Balla A. (2020). A 17-Gene Panel Genomic Prostate Score Has Similar Predictive Accuracy for Adverse Pathology at Radical Prostatectomy in African American and European American Men. Urology.

[356] Rayford W., Greenberger M., Bradley R.V. (2018). Bradley, Improving risk stratification in a community-based African American population using cell cycle progression score. Transl. Androl. Urol..

[357] Canter D.J., Reid J., Latsis M., Variano M., Halat S., Rajamani S., Gurtner K.E., Sangale Z., Brawer M., Stone S. (2019). Comparison of the Prognostic Utility of the Cell Cycle Progression Score for Predicting Clinical Outcomes in African American and Non-African American Men with Localized Prostate Cancer. Eur. Urol..

[358] Creed J.H., Berglund A.E., Rounbehler R.J., Awasthi S., Cleveland J.L., Park J.Y., Yamoah K., Gerke T.A. (2020). Commercial Gene Expression Tests for Prostate Cancer Prognosis Provide Paradoxical Estimates of Race-Specific Risk. Cancer Epidemiol. Biomark. Prev..

[359] Mohler J.L., Antonarakis E.S., Armstrong A.J., D’Amico A.V., Davis B.J., Dorff T., Eastham J.A., Enke C.A., Farrington T.A., Higano C.S. (2019). Prostate cancer, version 2.2019, NCCN clinical practice guidelines in oncology. J. Natl. Compr. Cancer Netw..

